# Weighted Stochastic Block Models of the Human Connectome across the Life Span

**DOI:** 10.1038/s41598-018-31202-1

**Published:** 2018-08-29

**Authors:** Joshua Faskowitz, Xiaoran Yan, Xi-Nian Zuo, Olaf Sporns

**Affiliations:** 1 Program in Neuroscience, Indiana University, Bloomington, IN USA; 2 Department of Psychological and Brain Sciences, Indiana University, Bloomington, IN USA; 3 Indiana University Network Science Institute, Indiana University, Bloomington, IN USA; 4 CAS Key Laboratory of Behavioral Science, Institute of Psychology, Beijing, China; 5 Research Center for Lifespan Development of Mind and Brain (CLIMB), Institute of Psychology, Beijing, China; 6 Key Laboratory for Brain and Education Sciences, Nanning Normal University, Nanning, Guangxi 530001 China

## Abstract

The human brain can be described as a complex network of anatomical connections between distinct areas, referred to as the human *connectome*. Fundamental characteristics of connectome organization can be revealed using the tools of network science and graph theory. Of particular interest is the network’s community structure, commonly identified by modularity maximization, where communities are conceptualized as densely intra-connected and sparsely inter-connected. Here we adopt a generative modeling approach called weighted stochastic block models (WSBM) that can describe a wider range of community structure topologies by explicitly considering patterned interactions between communities. We apply this method to the study of changes in the human connectome that occur across the life span (between 6–85 years old). We find that WSBM communities exhibit greater hemispheric symmetry and are spatially less compact than those derived from modularity maximization. We identify several network blocks that exhibit significant linear and non-linear changes across age, with the most significant changes involving subregions of prefrontal cortex. Overall, we show that the WSBM generative modeling approach can be an effective tool for describing types of community structure in brain networks that go beyond modularity.

## Introduction

The human brain forms a complex network of anatomically interconnected neurons and brain regions, the *connectome*^1^ that can be modeled and analyzed with the tools of network science and graph theory^2^. Modeling the brain as a network allows us to explore local as well as distributed properties of brain organization, using both descriptive^3^ and generative modeling approaches^4^. A hallmark of complex networks, including the human connectome, is the presence of subnetworks, also called communities or modules^5^. The set of communities that comprise a given network is referred to as the network’s community structure. This structure is useful for describing both large-scale and local patterns of the network^6^. At large-scale, we can measure differential connectivity trends between communities, e.g. across age^7^ or in relation to cognition^8^. Locally, we can use metrics such as the participation coefficient to assess node-wise aspects of the community structure^9,10^.

**Figure 1:**
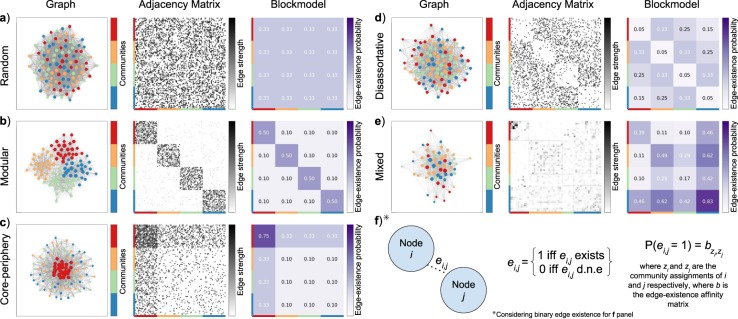
Three representations of network data: graph, adjacency matrix, block model. The graph is visualized as a force-directed^84^ graph layout, the adjacency matrix is visualized as a square matrix with entries for each edge between nodes, and the block model is visualized as a square matrix with entries for each edge-existence parameter between communities. (**a**) Random network (**b**) Modular network (**c**) Core-periphery network (**d**) Disassortative network (**e**) Mixed network, based on an example fit to brain network data of a single hemisphere (**f**) An illustration of a binary (unweighted) edge for each network data representation.

In many extant studies, network communities are operationalized as modular subnetworks, i.e. as groups of nodes that are more densely connected within, and more sparsely connected between groups. However, the process of identifying modules in networks, community detection, is an ill-defined problem with no universal definition^11–15^. Modular network communities are merely one plausible lens through which to analyze brain network communities. In fact, recent evidence demonstrates that the presence of diverse community structure connectivity patterns beyond modular configurations correlates with behavioral task performance^16^ For this investigation, we employ an alternative to the modularity approach by adopting a model from a family of methods called stochastic block models (SBM)^17–20^. The SBM splits nodes into blocks, within which all nodes are stochastically equivalent in terms of how they connect to the rest of the network. As a generative model, it has a well-defined likelihood function with consistent parameter estimates. It is also highly flexible, capable of modeling a wide variety of community structures, including the conventional modular, but also disassortative, core-periphery or mixed community structures (Fig. 1). Recent theoretical developments in SBM models have also enabled them to capture degree distributions^20^, overlapping communities^21^, and weighted edge weights^22,23^ as well as statistically principled model selection criteria^24,25^.

In this study, we employ the stochastic block modeling framework to analyze, cross-sectionally, how brain networks, and the community structure of these networks, are modulated across the human life span. Over the human life span the brain matures nonlinearly, from development to young adulthood, and into old age^26^. Notably, morphological changes in the cortical grey matter are heterogeneous, as spatially distinct regions of the cortex develop, mature, and decline at different time points and rates^27,28^. Additionally, the white matter architecture that supports connections between these distinct cortical regions develops at variable rates^29–31^. To characterize these changes in brain networks across the human life span several recent studies have applied tools of complex network analysis^7,32–35^. Using resting state functional connectivity MRI networks, studies have shown increases in connectivity between modules increases with age while connectivity within modules decreases^7,36^. The modularity of these networks has been shown to decease over the life span^35^. Concurrently, overall structural connectivity (total number of recovered streamlines) decreases as a function of age^7,37^, hypothesized to be a result of preferential detachment of short structural connections within modules^37^.

SBMs offer great flexibility as the way in which communities are defined transcends the narrower definition inherent in classical modularity maximization. Despite their methodological advantages, SBMs have only recently been applied to the analysis of brain networks^16,38–41^. Here we apply a weighted variant of the stochastic block model, called the Weighted Stochastic Block Model, or WSBM^22,23,42^, to whole-brain anatomical networks extracted from diffusion imaging and tractography data acquired across a major portion of the human life span. After designing a robust strategy for applying WSBMs to weighted connectome data, we fit WSBMs to group-averaged connectomes, as well as to individual connectome networks. We find patterns of age-related changes that unfold in specific sub-blocks of SBMs, representing bundles of connectome edges that exhibit significant linear or non-linear changes across the life span. We also demonstrate how to measure community structure change across age by conceptualizing community structure as a vector. Finally, we discuss the patterns of change we detected in this study in the context of previous work reporting on modularity and age-dependent changes in functional connectivity.

## Methods

### Data description

Our data was generated from 620 human subjects (63% female) from the enhanced Nathan Kline Institute-Rockland Sample (NKI-RS; fcon_1000.projects.nitrc.org/indi/enhanced/)^43^. Institutional Review Board approval was obtained for this project at the Nathan Kline Institute (#226781 and #239708) and at Montclair State University (#000983 A and #000983B) in accordance with relevant guidelines. Written informed consent was obtained for all study participants. Written consent and assent was also obtained from minor/child participants and their legal guardian. In the present study, human data used was de-identified and provided open-access via an Amazon S3 Bucket (fcon_1000.projects.nitrc.org/indi/enhanced/neurodata.html). The NKI-RS dataset is a cross-sectional community sample that covers a wide range of the human life span (6–85 years old; std. dev: 20.88). Both T1-weighted (T1w) and diffusion (dMRI) images were collected for each study participant on a 3 T Siemens Magnetom Tim Trio scanner (Siemens Medial Solutions USA: Malvern PA, USA) using a 12-channel head coil. T1-weighted magnetization rapid acquisition gradient-echo (MPRAGE) were acquired with the following scan parameters: echo time (TE): 2.52 ms; repetition time (TR): 1900 ms; flip angle (FA): 9 degrees; FOV: 176 sagittal slices at 250 × 250 mm, with 1 mm spacing; GRAPPA acceleration factor of 2; acquisition time: 4:18 min. DWI were acquired with the following scan parameters: TE: 85 ms; TR: 2400 ms; FA: 90 degrees; FOV: 64 axial slices of 212 × 212 mm, with 2 mm spacing; multi-band acceleration factor: 4; 128 directions in single-shell; b-value 1500 s/mm^2^; 9 non-weighed diffusion volumes; anterior» posterior phase encoding direction; acquisition time: 5:58 min. 671 dMRI datasets were initially downloaded. Data exclusions included: 13 subjects did not have viable T1w (FreeSurfer failure); 12 dMRI were visually judged as having image artifacts (based on viewing medial axial, coronal, and sagittal slices of fractional anisotropy map); 24 tractography reconstructions were visually judged as poor (~3.8% of tractographies generated; based on 6 rotated views of the tractogram and looking for areas of non-smooth streamline paths); 2 streamline count matrices were labeled outliers based on an edge density cutoff (sparsity z-scores of -4.1 and -5.6)—adjacency matrices that failed to reach a binary edge density of 25% were deemed too sparse.

### MRI pre-processing

T1w images were run through FreeSurfer’s (surfer.nmr.mgh.harvard.edu) *recon-all* pipeline to obtain a cortical surface reconstruction and surface mapping to the FreeSurfer *fsaverage* space. We reconstructed the Yeo17 network parcellation^44^ (114 cortical nodes, source: github.com/ThomasYeoLab) in the T1w native space using FreeSurfer’s nonlinear surface warps. We then applied FSL *fast*^45^ to the skull-stripped T1w to obtain grey matter (GM), white matter (WM), and cerebral spinal fluid (CSF) partial volume estimation (PVE) maps. The bias-corrected T1w were rigidly aligned to MNI 152 1 mm isotropic space using FSL *flirt*. All parcellations and PVE maps were aligned to MNI space by applying the *flirt* linear transformation. PVE maps were thresholded at 0.5, to obtain maps of the majority volume estimates for each voxel.

We first denoised the dMRI using a spatially adaptive denoising algorithm^46^. dMRI were then corrected for motion using FSL *eddy_correct*, with the normalized mutual information cost metric. The average unweighted diffusion volume (B0 image) was then linearly aligned to the T1w in MNI 152 2 mm space using the FSL *flirt* boundary-based registration routine^47^. The inverse of this transformation was applied to the T1w to bring the T1w into the dMRI native space. We then used ANTs SyN registration^48^ to nonlinearly correct the dMRI for eddy current distortion in the phase encoding direction. The dMRI images were finally aligned to MNI 152 2 mm space by concatenating and applying the *eddy_correct*, ANTs warp, and *flirt* transformations, to interpolate the dMRI only once. dMRI b-vectors were rotated accordingly.

We generated streamline tractography in the MNI 152 2 mm isotropic space using Dipy^49^. We first modeled the fiber orientation distribution function (fODF) at each voxel using constrained spherical deconvolution^50^; fit using a recursive calibration^51^ and a spherical harmonic order of 8. We placed 9 random seeds in each voxel of a white matter mask, generated by calculating the intersection of the PVE WM and FreeSurfer WM segmentation. We used Dipy’s *LocalTracking* module to deterministically propagate streamlines bidirectionally from each seed. Streamlines were generated at 0.5 mm steps, with a max turning angle of 30 degrees. Streamlines longer than 5 mm and terminating in the GM PVE map, while avoiding the CSF PVE map^52^, were retained.

We constructed streamline count adjacency matrices by counting the number of streamlines that terminated in each region of interest (ROI) of the Yeo network parcellation. We disregarded nodes connected by only one streamline as noise and set these entries in the count matrix to zero. We recorded the voxel volume of each ROI of the Yeo parcellation and normalized the streamline count matrices by geometric mean volume of each pair of connected ROIs. This step was taken to remove potential edge-weight bias for larger ROIs. Hence, we recorded the weights of our structural connectivity matrices (connectomes) as streamline density measurements, as in previous studies^3,16,53^.

### Community detection with the stochastic block model

Communities described by the SBM, also called blocks, are groups of nodes that are stochastically equivalent. Hence, nodes in the same community connect to all other nodes with a similar pattern. An SBM block does not require nodes within a block to connect densely to each other, and sparsely to other blocks. Rather, the probability at which nodes in a block connect to other nodes in the same block is a parameter with the same importance as all other block interactions. For classic SBM, the probability of an edge existing between two nodes of block A and block B will be described by a Bernoulli distribution with parameter *theta* that describes the probability of an edge existing between any two nodes of block A and block B. With an SBM of *k* blocks, we can build a *k* × *k* affinity matrix *b* that describes the probability of a connection (edge-existence) between nodes of each block based on the Bernoulli distribution parameterized by the corresponding entry in affinity matrix *b* (see Fig. 1, panel f). The between block edge-existence parameters describe the connectivity of each node to each block independently.

Recently, the SBM has been extended to model networks with weighted edges, referred to as the weighted stochastic block model, or WSBM^22,23,42^. With this advancement, we can apply the SBM framework to weighted networks commonly encountered in the network neuroscience literature. Using openly-available code (tuvalu.santafe.edu/~aaronc/wsbm/)^23,42^, we fit the WSBM to structural connectivity matrices using a variational-Bayesian approximation approach. For our application, we chose to use the WSBM described by the following generative steps:

- For each node, assign a community membership
- For each pair of communities, assign edge-existence and edge weight parameters
- For each edge, draw from the Poisson distribution with the corresponding edge-existence parameter
- For each existing edge, draw from the normal distribution with the corresponding edge weight parameters

### Community structure fitting workflow

**Figure 2:**
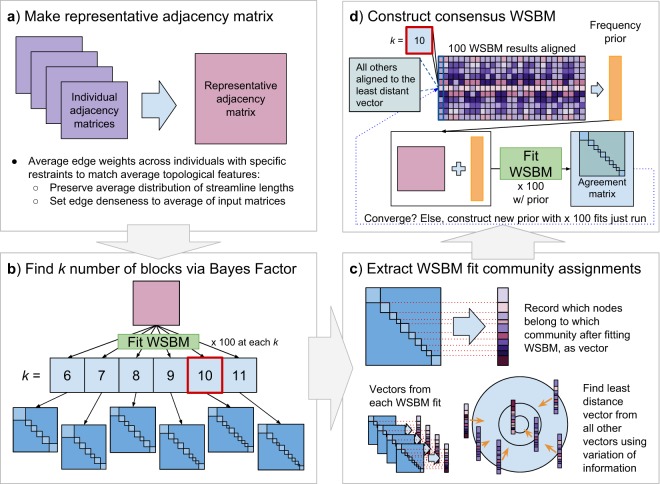
Creation of consensus WSBM model. (**a**) A representative adjacency matrix was constructed by averaging across multiple individual matrices. This averaging was performed with specific restraints to mitigate bias. (**b**) The number of blocks must be specified a priori; to identify an appropriate number of blocks (*k*), the WSBM was fit 100 times at *k* = 6, 7…11. We recorded the average log-evidence of 100 model fits at each *k* and used Bayes factor to determine *k* in a data-driven manner. (Note that the red box indicates the *k* = 10 parameter identified in this study) (**c**) At the *k* with the highest likelihood, for each of the 100 fits, we recorded the community assignment for each node as a vector. We then computed the least distant community assignment vector from the 100 fits. (**d**) Aligned results of the 100 fits were averaged to create a community assignment prior. This process was repeated until a convergence criterion was met.

Fitting the WSBM on connectome data yields stochastic results (like other community detection algorithms such as modularity maximization). When comparing across different community structures, the correspondence between specific communities could become unclear. Therefore, we first sought to create a representative WSBM partition of our data, to serve as a generalizable model to provide an initial overview of network structure in a specific age window, and to seed further age-dependent analysis. To create a representative matrix to infer the community structure of, we aggregated adjacency matrices from 53 young adult subjects (25–35 years old; 51% female) and averaged the data subject to several constraints (Fig. 2, panel a)^16,54^. Specifically, the edge density of the representative matrix was set to match the average edge density of the input sample. Additionally, the distribution of streamline lengths across the input matrices was maintained, to mitigate bias against hard-to-recover long streamlines^55^.

Fitting WSBM to data requires searching over a large parameter space. Instead of using ad-hoc greedy heuristics, we adopt a multi-step fitting scheme. The first level of fitting involves fitting the model with a uniform prior, to broadly search the parameter space of plausible block partitions. We inferred WSBM structure with uniform block assignment prior parameters for 250 independent trials of the inference process. At each trial, the uniform block assignment is used to seed an expectation maximization algorithm^42^ to fit the following WSBM model parameters to the observed data. After 250 trials, the most likely model fit is retained (maximum posterior) and the most likely block assignments for each node are recorded. The second level of fitting involves fitting the model with increasingly stronger priors. The previously inferred block assignment was used to construct a biased block assignment prior parameter. That is, for each node, we assign a 100% higher likelihood for that node to be assigned to the previous most likely block and assign uniform likelihood to the other (*k*-1) blocks. With this prior, we ran 100 more independent trials. We iterated this second stage 10 times, incrementing the concentration of the most likely nodal prior assignment by 1.5 times (150%, 200%, … 600%).

We chose the number of communities (*k*) after repeatedly fitting the WSBM at each value of *k* = 6, 7 … 11 (Fig. 2, panel b). We fit the WSBM 100 times at each *k* and recorded the marginal log-likelihood (which penalizes model complexity, ensuring that we do not fit the data better simply by increasing the number of parameters) at each fit. Using Bayes factors, we compare the partitions via the difference in marginal log-likelihood of each model fit^42^.

Next, we sought to derive a Bayesian consensus WSBM from multiple fits of our data^56^. First, we aggregated the results of the 100 WSBM fits at our data-driven selected *k*. Next, we choose a representative partition from these 100 fits by determining the partition least distant from all other partitions (centroid; Fig. 2, panel c). To do this, we computed the pairwise distance between all 100 partitions using variation of information (VI)^9,57^. By summing across the rows of this distance matrix, we selected the partition that was least distant (minimum sum) from all others^58^. We then aligned the remaining 99 partitions to the reference partition via the Munkres algorithm^59^. The aligned partition matrix (size: 114 × 100 [nodes × number of fits]) was used to make a new nodal assignment prior, based on the frequencies a node was assigned to each of the *k* communities across 100 fits. This new prior was used as input for 100 more WSBM fits (Fig. 2, panel d). This process was repeated until a convergence criterion was met^60^.

We also created an alternative modular community structure to compare against. To match the number of modular communities to the number of communities learned in the WSBM consensus model, we ran the deterministic spectral modularity maximization algorithm for weighted data implemented in the Brain Connectivity Toolbox (sites.google.com/site/bctnet/; function *modularity_und*)^61^ across a range of gamma values (gamma: 0.5 to 4.0, at 0.01 steps). This resulted in 351 modular partitions with varying numbers of communities. We identified all partitions with an equal number of communities to the WSBM consensus model and from these partitions we selected one least distant to the WSBM consensus model (measured by VI).

Finally, we used the consensus community structure models to seed community structure fitting on individual-level brain network data. The WSBM was fit to each subject’s adjacency matrix with a prior community affiliation based on the WSBM consensus model concentrated at a level of 3. Thus, for each node, the community assignment of that node in the prior was 3 times more likely than an assignment to any of the other *k*-1 communities. The WSBM fitting procedure was then conducted as described previously. We collected five independent WSBM fits for each subject and retained the centroid partition of these fits. We also extracted a modular partition for each subject by running the spectral modularity maximization for weighted data, sweeping over levels of gamma from 0.5 to 4.0 in 0.01 increments. We identified modular partitions with *k* communities and retained the partition closest to the WSBM consensus model as measured by VI, to facilitate unbiased comparison. There is no guarantee that a modular partition with *k* communities will result from our sweep across gamma values. We excluded subjects for which we did not find a modular partition of *k* communities from the subsequent individual fits analysis (29 subjects).

### Analysis methods

To assess how well our models fit the empirical data, we followed a generative model evaluation framework^4^. We generated synthetic data using the inferred edge-existence and edge weight parameters of the WSBM consensus model. To create a comparable generative model from the modular partition, we used the tools of the WSBM fitting toolbox to fit the modular structure with an absolute prior (100% and 0% probabilities), and thus, did not perform model inference. We generated 10,000 synthetic adjacency matrices from both models. At each iteration, we recorded four binary network statistic distributions of the synthetic data: degree (*d*), clustering coefficient (*c*), betweenness centrality (*b*), and node Euclidean distance (*e*). We compared each synthetic statistic distribution with the empirical distribution of that statistic from the representative adjacency matrix (the data the model was derived from) using the Kolmogorov–Smirnov (KS) statistic, which measures the maximum difference between two empirical cumulative distribution functions. We computed the average KS statistic and conceptualized this as the energy of the synthetic network compared to the empirical network^4^.

$$KS\,Energy=mean(K{S}_{d},K{S}_{c},K{S}_{b,}K{S}_{e})$$

Lower energy indicates a synthetic network with network statistic distributions that more closely resemble the empirical network statistic distributions. We define energy here as mean KS as opposed to maximum KS, as in^4^, so as not to bias the result by any one statistic that might produce systematically higher KS. In the previous study, the max KS was desirable because this metric was used for further optimization of the model. Here, our goal was to measure model performance, without changing the inferred model parameters.

We also sought to evaluate whether the set of inferred parameters of the generative models was meaningful for reducing the energy of the generated synthetic matrices. It could be that the mere modeling of distributions between blocks, regardless of the parameters, would be sufficient to generate synthetic networks with low energy. To test this, we generated 10,000 synthetic adjacency matrices and randomly permuted the intact models’ parameters of the edge-existence and edge weight distributions at each iteration.

Next, we wanted to evaluate the extent to which each community structure preserved hemispheric symmetry. We proceeded under the assumption that at this level of analysis, it would be plausible to expect to find homotopic organization^34^; that is, that large patterns of organization of the right and left hemisphere organization should appear similar. To measure how the community structure captures laterality, we measured three weighted community-based network statistics: participation coefficient, within-community z-score, and assortativity. These three network measurements produce node-wise statistics that are relative to a given community structure. We measure each statistic given each consensus partition in each of the 620 subjects. For each subject, we compute the KS statistic between the left and right hemispheric distributions of the community-based network statistics.

We evaluated how the connectivity patterns between communities change over time. Our first approach involved measuring the total edge weight between communities. For each of *N* subject’s brain network data, we created a *k* × *k* block matrix recording the total weight between each community of the community structure being analyzed. As our brain network data are symmetric, we analyzed the upper triangle plus main diagonal of the block matrix, totaling (*k*^2^ − *k*)/2 + *k* tests. To examine age-related trends in in the values of these block strengths, we employed a multiple linear regression (MLR) analysis to model block strength as a linear combination of predictors. The MLR model was formulated as one of three models: (1) linear, (2) quadratic curve, (3) Poisson curve (as in^31^):

1 $$y={\beta }_{0}+{\beta }_{1}\times age+{\beta }_{G}\times G+\varepsilon $$

2 $$y={\beta }_{0}+{\beta }_{1}\times age+{\beta }_{2}\times ag{e}^{2}+{\beta }_{G}\times G+\varepsilon $$

3 $$y={\beta }_{0}+{\beta }_{1}\times age\,\times \,{e}^{-{\beta }_{2}\times age}+{\beta }_{G}\times G+\varepsilon $$

where *y* is the dependent variable, in this case a vector [number of measurements (*n*) × 1] of block strengths between communities *i* and *j*, *β*_1_ and *β*_2_ (if necessary) are weights estimated by ordinary least squares regression, *G* is a matrix [*n* × 2] nuisance covariates (sex, total network strength) with *β*_*G*_ [*n* × 2] also representing weights estimated by ordinary least squares regression, and *ε* is a vector [*n* × 1] of residual error. We also conducted tests with an additional nuisance parameter indexing motion across the DWI acquisition, which would make *G* and *β*_*G*_ size [*n* × 3]. We implemented the MLR by first linearly regressing out the nuisance covariates from the covariate of interest and using the residuals to regress against age. We fit each MLR model to each block strength vector and calculated model accuracy using leave-one-out cross validation (LOOCV). We calculated the root mean-squared-error (RMSE) of each fit and chose the model (linear, quadratic, Poisson) with the lowest error. To obtain a p-value, we randomly permuted age across 10,000 least-squares fit iterations. We retained trends with a computed p-value that passed Bonferroni correction for 55 comparisons (α = 0.0009). For each MLR model we report the coefficient of determination (*R*^2^) calculated from the LOOCV procedure^29^:

$${R}^{2}=1-\frac{{\sum }_{i=1}^{n}{({y}_{i}-{y^{\prime} }_{i})}^{2}}{{\sum }_{i=1}^{n}{({y}_{i})}^{2}}$$

where *n* is the number of measurements, *y* [*n* × 1] is vector of dependent variables, and *y′* [*n* × 1] is the vector of model predictions.

For our second approach to assess how overall community structure changes across age, we pursued mathematical comparisons that measured all community interactions simultaneously. To do this, we utilized the block matrix, which is a *k* × *k* matrix, in which each entry *i,j* is a measure of the edges (e.g. total strength, average strength) between communities *i* and *j*. Thus, a block matrix provides a condensed information about network connectivity, given a community structure. We unrolled the upper triangle plus main diagonal of the average block matrix into a vector of length $l=\frac{{k}^{2}-k}{2}+k$. We then used this vector as an *l* dimensional representation of the overall pattern of community structure and measured these vectors similarity and distance to our consensus models. For this analysis, we used the block matrix of average strength between communities, as opposed to total strength as in the previous analysis, to mitigate the effect of community size for this analysis.

We measured how similar/distant each subject’s community structure vector was to the consensus model community structure vector. In the WSBM evaluation, we used the affinity parameters of the WSBM consensus model to obtain the WSBM consensus model vector. For the modular evaluation, we measured the empirical block matrix based on the modular fit to the representative adjacency matrix to obtain the modular consensus model vector. For each of *N* subjects, we unrolled the upper triangle (including diagonal) of an average strength block matrix given each partition. We compared each of *N* community structure vectors to the consensus model vector using cosine similarity and city block distance^62^. We then used the MLR scheme detailed previously to assess the proportion of the variance in subject-level vector similarity/distance that is due to age.

To analyze individually fit community structures, we employed the previously described vector comparison scheme with individually fit community structures. We recorded the distance of individual vectors to the model prediction vectors, as described previously and employed MLR to measure trends in age versus subject-level vector similarity/distance.

Additionally, we measured nodal versatility across individually fit partitions^63^. Nodal versatility is an index of how consistently nodes are classified in the same community across repetitions of a community detection algorithm. We used this measure to obtain a versatility index for each of the 114 nodes in use. Instead of measuring nodal versatility across repetitions of an algorithm, we measured the node versatility across subjects to evaluate differences in community detection techniques. We employed a permutation test to create a distribution of null versatility differences to test for statistical significance. If there is no difference between the methods, exchanging subject A’s WSBM community vector for subject A’s modular community vector would not affect the node versatility index. Therefore, for each permutation we constructed two complementary node × subject matrices, in which we shuffled the type of community vector included. We computed the node versatility of each node × subject matrix and took the node versatility difference at each node. We measured the empirical versatility difference against the null distribution of versatility differences at each node to obtain a p-value, and report nodes that pass Bonferroni correction for 114 comparisons (α = 0.0004).

## Results

### Model fitting workflow

Our consensus fitting procedure was intended to aggregate the results of 100 WSBM model fits. We found that our method was consistent despite internal stochastic elements of the code; if given the same data, the process produced the same output in each of 20 repetitions. We also ran the process on 100 additional independent runs at *k* = 10 to create a new frequency prior and observed that the resulting community structure had a 0.80 normalized mutual information (NMI) to the obtained WSBM consensus model. As a final test, we bootstrap-sampled the results of the 100 fits to create different initial frequency priors. Comparing the results of this process with the obtained WSBM consensus model resulted in NMI measurements with a mean of 0.97 (±0.04, range = 0.86–1.0).

### Consensus community structure

**Figure 3:**
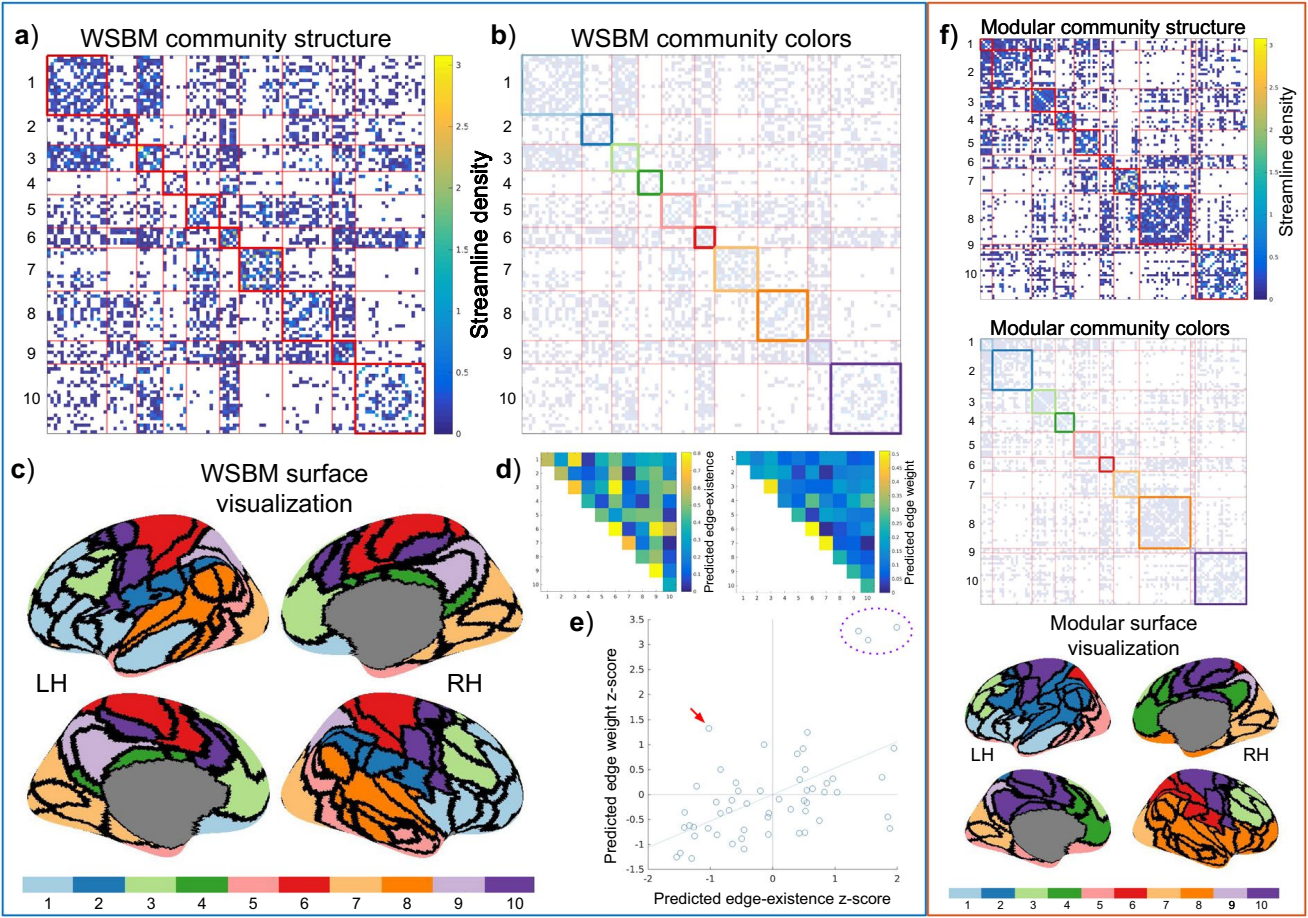
The WSBM consensus model, fit to a representative matrix averaged across 53 young adult subjects. LH = left hemisphere; RH = right hemisphere. (**a**) The adjacency matrix ordered by the blocks of the WSBM consensus model. On-diagonal blocks are outlined in red, off-diagonal blocks are outlined in light-red. (**b**) The adjacency matrix of the young adult data with the on-diagonal blocks colored to match the inflated surface view (in panel c). (**c**) The community structure of the consensus model visualized on the inflated surface of the left and right hemispheres. (**d**) The predicted edge-weight and edge-existence matrices; the entries of these matrices contain the consensus model predictions for the average edge-weight and edge-existence for each block interaction. To calculate the consensus model’s average block interaction prediction, these two matrices can be multiplied element-wise. (**e**) We plot the paired parameters of the block interactions (z-score transformed). From this plot, we observe a general linear relationship between predicted edge-existence and predicted edge weight for each block interaction. We highlight how the WSBM fit densely connected and densely weighted areas (purple dotted circle) as well as non-modular block interactions (red arrow). (**f**) Visualization of alternative modular community structure, visualized as adjacency matrices and on the cortical surface in the same manner as WSBM model. The labeling of these alternative community structures (represented as colors) are aligned to closely match the labeling of the WSBM model using the Munkres algorithm.

Using the consensus method outline above, we inferred WSBM community structure from the representative brain network data (edge-existence density = 30.1%). We identified 10 bilateral communities with mixed topology profiles and attributes (Table 1). Sizes of the communities ranged from 6 to 21 nodes. The WSBM model estimates parameters that govern the distribution of edge and edge weight between communities; these parameters are visualized in panels d and e of Fig. 3. Generally, the WSBM modeled a positive relationship between edge existence and edge weight; in other words, block interactions with low edge existence were modeled with low edge weight or high edge existence with high edge weight. One block interaction, 4–10, stands out as having been modeled with a low edge existence but high edge weight (red arrow, panel e, Fig. 3). This interaction is predicted to be connected at a probability of 13% and its edges are predicted to have a strength (average streamline density) of 0.29. Three block interactions are notable for having high edge weight and edge-existence parameters: 3–3, 6–6, and 7–7. In the modular community structure, not all communities identified were bilateral; that is, some communities were confined to only one hemisphere. The modular communities range in size from 2–22 nodes. Measuring the total Euclidean distance between nodes of communities in each partition, in each subject, we find that the modular community structure consists of communities much more spatially compact (M = 8.29 × 10 ^3^mm, SD = 89.01) than the WSBM partition (M = 8.68 × 10 ^3^mm, SD = 62.54 mm; two-tailed paired t-test with unequal variances; t(1.11 × 10^3^) = 88.35, p < 10^−9^).

**Table 1:** Table of community statistics for the WSBM and modular consensus partitions. Statistics from the representative young adult matrix; across-subject mean ± standard deviation in parentheses.

Community Label	Mean within-community strength	Mean between-community strength	Mean community participation coef.	Community assortativity
**WSBM**				
1	0.11 (0.096 ± 0.018)	0.042 (0.04 ± 0.0066)	0.77 (0.73 ± 0.027)	−0.035 (−0.027 ± 0.025)
2	0.095 (0.08 ± 0.027)	0.035 (0.032 ± 0.0076)	0.82 (0.76 ± 0.022)	0.011 (−0.006 ± 0.032)
3	0.33 (0.28 ± 0.076)	0.054 (0.05 ± 0.0093)	0.78 (0.75 ± 0.022)	0.19 (0.16 ± 0.078)
4	0.051 (0.046 ± 0.029)	0.038 (0.033 ± 0.0086)	0.73 (0.64 ± 0.054)	−0.084 (−0.084 ± 0.054)
5	0.11 (0.12 ± 0.032)	0.035 (0.034 ± 0.006)	0.79 (0.73 ± 0.027)	0.032 (0.034 ± 0.033)
6	0.4 (0.37 ± 0.12)	0.056 (0.051 ± 0.009)	0.78 (0.75 ± 0.025)	0.26 (0.24 ± 0.13)
7	0.33 (0.33 ± 0.049)	0.021 (0.022 ± 0.0041)	0.48 (0.45 ± 0.05)	0.25 (0.24 ± 0.055)
8	0.11 (0.1 ± 0.021)	0.033 (0.032 ± 0.0067)	0.77 (0.71 ± 0.03)	0.029 (0.026 ± 0.022)
9	0.19 (0.19 ± 0.098)	0.052 (0.049 ± 0.0086)	0.85 (0.79 ± 0.032)	0.098 (0.082 ± 0.094)
10	0.084 (0.063 ± 0.017)	0.032 (0.027 ± 0.0046)	0.7 (0.66 ± 0.03)	−0.0068 (−0.018 ± 0.019)
**Modular**				
1	0.2 (0.19 ± 0.057)	0.05 (0.048 ± 0.0095)	0.81 (0.75 ± 0.028)	0.066 (0.051 ± 0.058)
2	0.11 (0.1 ± 0.024)	0.034 (0.03 ± 0.0059)	0.7 (0.63 ± 0.039)	0.015 (0.018 ± 0.028)
3	0.3 (0.25 ± 0.061)	0.042 (0.041 ± 0.0083)	0.72 (0.68 ± 0.034)	0.21 (0.15 ± 0.069)
4	0.27 (0.23 ± 0.07)	0.034 (0.033 ± 0.0073)	0.7 (0.66 ± 0.051)	0.19 (0.14 ± 0.075)
5	0.19 (0.19 ± 0.035)	0.043 (0.041 ± 0.0084)	0.71 (0.66 ± 0.04)	0.062 (0.059 ± 0.049)
6	0.19 (0.18 ± 0.063)	0.046 (0.042 ± 0.011)	0.78 (0.7 ± 0.044)	0.049 (0.05 ± 0.073)
7	0.35 (0.35 ± 0.055)	0.025 (0.026 ± 0.0055)	0.52 (0.49 ± 0.042)	0.28 (0.27 ± 0.061)
8	0.14 (0.14 ± 0.024)	0.029 (0.028 ± 0.0061)	0.57 (0.52 ± 0.055)	0.068 (0.058 ± 0.027)
9	0.5 (0.49 ± 0.48)	0.057 (0.054 ± 0.015)	0.87 (0.81 ± 0.036)	0.35 (0.34 ± 0.45)
10	0.14 (0.1 ± 0.019)	0.026 (0.025 ± 0.0042)	0.55 (0.55 ± 0.038)	0.085 (0.044 ± 0.023)

We also measured how the high strength nodes were distributed amongst communities in the different community structure models. We recorded in which communities the top 25%-degree (binary degree and weighted degree) nodes for each subject appeared and then measured how consistently these high strength nodes were dispersed among the communities across subjects (Table 2). We find that the WSBM model most consistently groups high degree nodes in a similar pattern across subjects, as measured by the intraclass-correlation coefficient, *ICC*(3,1)^64^.

**Table 2:** How consistently high strength nodes (top 25%) appear in the same community, measured across subject with the intraclass correlation coefficient; confidence interval computed with 500 bootstrap iterations.

Community structure model	Binary degree ICC (95% confidence interval)	Weighted degree ICC (95% confidence interval)
WSBM	0.83 (0.82–0.84)	0.74 (0.73–0.75)
Modular	0.64 (0.62–0.65)	0.59 (0.58–0.61)

### Model fitting comparison

**Figure 4:**
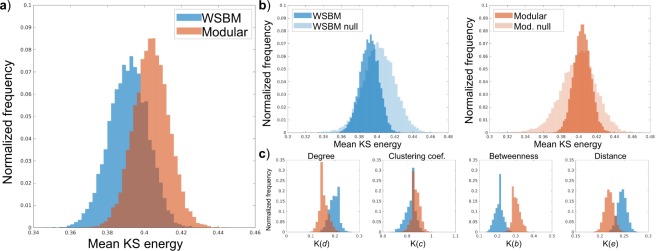
Comparison of WSBM and modular generative capabilities and characteristics. (**a**) We compared the mean KS energy between generated synthetic data based on the community structure models and empirical data; we observe that the WSBM generates synthetic data with a lower mean KS statistic—demonstrating that WSBM synthetic networks have network statistic distributions more representative of the empirical data. (**b**) We compare each model energy distribution with the energy distribution from a randomized model containing the same affinity parameters; we observe that the mean energy of the WSBM model is significantly lower than the WSBM randomized model; we observe that the mean energy of the modular model is higher than the modular randomized model (**c**) We show the KS statistics of each network statistic that comprises the KS energy formulation.

We found significant differences between the community structures identified through WSBM inference and modularity maximization. The mean generative energy of the WSBM model (M = 0.391, SD = 0.011) was significantly lower than the mean of the modular model (M = 0.404, SD = 0.009) (two-tailed paired t-test with unequal variances; t(1971) = −88.03, p < 10^−9^). This WSBM generative energy distribution had a significantly lower mean than the mean of the randomized WSBM generative energy distribution (M = 0.402, SD = 0.377; t(1630) = 52.16, p < 10^−9^) whereas the mean of the randomized modular generative energy distribution (M = 0.400, SD = 0.360) was lower than its distribution from the intact model (t(1443) = −17.68, p < 10^−9^). The individual distributions averaged over to calculate generative energy are also shown in panel c of Fig. 4.

**Figure 5:**
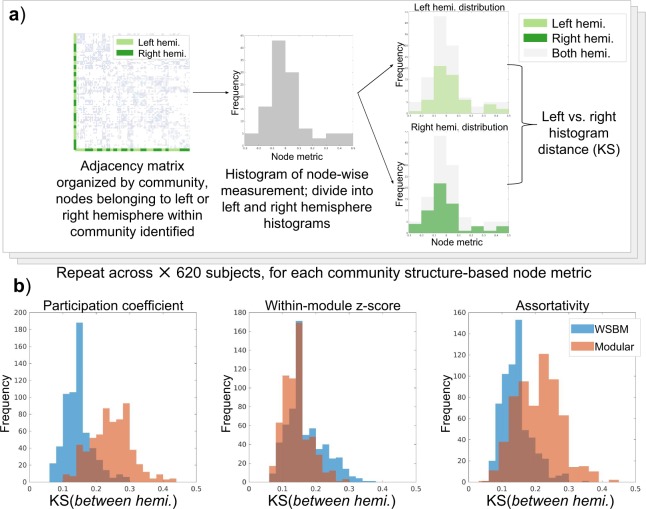
Evaluation of the laterality of the WSBM and modular community structures. (**a**) We compute community-based network statistics on the subject network data, yielding node-wise statistics; we then calculate separate distributions of these statistics based on each node’s laterality; we then measure the distance between these two distributions (note the shaded grey distribution illustrates the full, bilateral network statistic distribution) (**b**) We compare the laterality of each community structure by illustrating the KS between hemispheric distributions of community-based network statistics. We observe that the WSBM partition balances the participation coefficient and node-wise assortativity distributions across hemispheres better than the modular partition. The modular partition preserves within-community z-score better than the WSBM partition.

We studied how the community structures under investigation capture symmetric network structure across hemisphere, as measured by node-wise network statistics (Fig. 5). We measured the histogram distances between the left and right hemisphere histograms of participation coefficient, within-community z-score, and assortativity using the KS statistic. We found the mean of participation coefficient and node-assortativity KS distributions to be significantly lower for the WSBM community structure compared to the KS distribution for the modular community structure. Statistical comparisons are shown in Table 3.

**Table 3:** Statistical comparisons between community structure-based node statistics.

Node-wise network statistic	Mean ± standard deviation	t-statistic	p-value
WSBM participation coefficient	0.14 ± 0.04		
Modular participation coefficient	0.24 ± 0.06		
		t(1128) = −34.10	p < 10^−9^
WSBM within-module z-score	0.17 ± 0.06		
Modular within-module z-score	0.14 ± 0.04		
		t(1139) = 9.24	p < 10^−9^
WSBM assortativity	0.14 ± 0.05		
Modular assortativity	0.2 ± 0.07		
		t(1107) = −20.61	p < 10^−9^

### Changes across the life span

**Figure 6:**
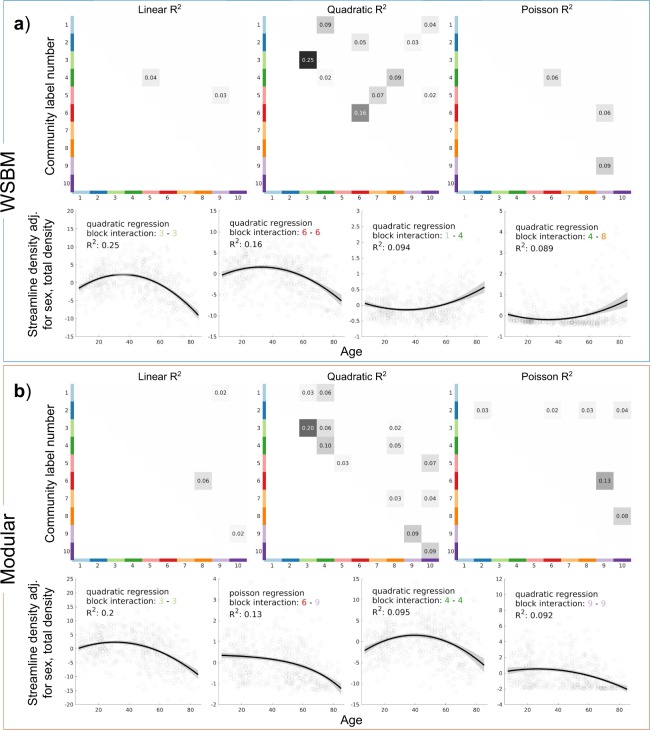
Results of multiple linear regression analysis on edge strengths of community interactions in the WSBM (**a**) and modular community (**b**) structures. The strongest quadratic relationship between age and community interaction edge strength is the 3–3 block interaction. All community interaction regressions that are statistically significant are shown. The top 4 MLR trends are visualized for each community structure model; the bootstrapped 95% confidence interval is shaded in grey.

We used multiple linear regression (MLR) to measure trends of block interaction strength across age. We observe strong quadratic relationships for within-community trends for both the WSBM and modularity models. We report the top 4 MLR trends for each model in Fig. 6, and the top 12 trends for each model in Fig. S3. The inverted-U shaped quadratic trend for block interaction 3–3 had the largest *R*^2^ for both the WSBM and modular partitions (*R*^2^: 0.25 and 0.20 respectively). Community *six* was involved in the second strongest trends for both the WSBM and modular partitions. Fewer on-diagonal MLR trends were significant for the WSBM partition than for the modular partition (4 and 6 block interactions respectively).

**Figure 7:**
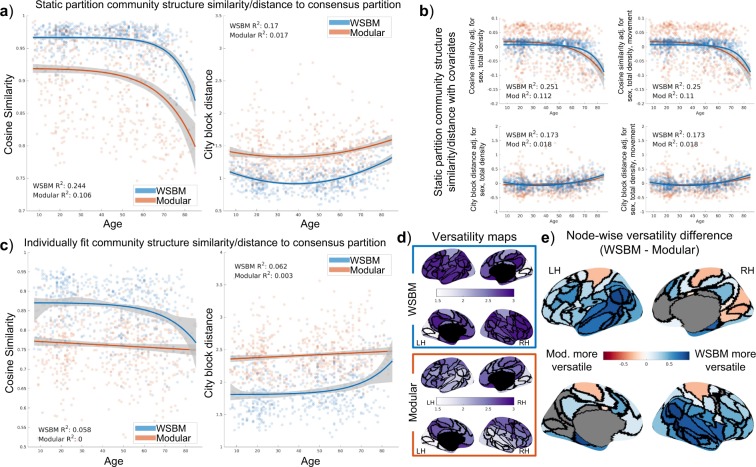
Measuring community structure vector similarity/distances. (**a**) Regression of age versus vector measurements using the static community structure measured on subject-level data. Bootstrapped 95% confidence interval of trend shaded in grey. (**b**) Regression of age versus vector distance adjusted for covariates. The trends do not change with the adjustment for covariates. (**c**) Regression of age versus vector measurements of individually fit community structure to the consensus partitions. (**d**) Maps displaying across subject nodal versatility for WSBM and modular fit partitions. (**e**) Map of versatility differences between the two methods. In the difference map, only nodes with statistically significant differences are colored.

When measuring community structure as a vector and computing vector similarity and distance from each subject to a consensus partition, we observed differences based on community partition used and observed strong MLR trends across age. Cosine similarity between subject and WSBM consensus vectors (M = 0.96, SD = 0.027) was significantly higher than the cosine similarity between subject and modular consensus vectors (M = 0.92, SD = 0.066; two-tailed paired t-test with unequal variances; t(823.75) = 20.46, p < 10^−9^). Using the city block distance measurement also displayed a significant overall difference (t(960.89) = −24.44, p < 10^−9^) between using the WSBM (M = 1.00, SD = 0.19) or modular vectors (M = 1.38, SD = 0.34). These trends did not change substantially after regressing out covariates (sex, total network strength, movement; Fig. 7 panel b).

Individual vector similarity between subject and consensus partition varied strongly with age using the WSBM and modular consensus vectors. When measuring the similarity of subject to WSBM vector across age, we observed a Poisson curve with an *R*^2^ of 0.24. For the analogous trend using the modular vector, we observed a Poisson curve with an *R*^2^ of 0.11. The asymmetry of the Poisson curve allowed a fit to the data that suggested a pattern of high similarity between individual and consensus partition from childhood through approximately age 60, followed by a steep decline. When measuring the distance between subject and consensus vector across age, we observe in both the WSBM and modular cases a U-shaped trend (WSBM *R*^2^: 0.17; modular *R*^2^: 0.02).

When measuring individually-fit community structure vectors to each consensus vector, we again find that the WSBM derived community structure vectors are both more similar (t(1.20 × 10^3^) = 22.07, p < 10^−9^) and less distant (t(1.12 × 10^3^) = −25.84, p < 10^−9^) than the modular consensus vectors. We find that using the WSBM fit explains more of the variance in vector similarity than using the modular fit. However, the MLR trend was weak for regressions of both vector distances versus age (*R*^2^: 0.06 for both cosine similarity and city block distance). Using the modular fit, we observe that trends that do not, or negligibly, explain the variance in vector similarity or distance (*R*^2^: 0 cosine similarity and *R*^2^: 0.01 for city block distance).

We recorded the versatility at each node for each community detection method. We observed that nodal versatility across nodes is higher when using the WSBM method (M = 2.66, SD = 0.32) compared to modularity maximization (M = 0.32, SD = 0.31; two-tailed paired t-test with unequal variances; t(225.89) = 8.40, p < 10^−9^). The pattern of node versatility differences (range: −0.35–0.95) shows differential influence of spatial proximity between the community detection methods. We note that along the temporal lobe nodes reach the largest difference between the two methods (right temporal-occipital node), with a mean difference of 0.75 in nodes labeled “temporal”.

### Additional parcellation analyses

We also inferred WSBM and modular consensus community structures using an alternative parcellation scheme, based on anatomical node definitions^3^. We report results of these evaluations in the Supplementary Information and show converging results with the analysis performed using the Yeo parcellation (Figs S4–S6). Additionally, we evaluated the degree of spatial similarity between community structures across parcellation selection. We find that both the WSBM and modular partitions across parcellation selection are statistically similar, compared to randomized community structures^65,66^.

## Discussion

Communities in brain networks have been hypothesized to form “building blocks” of the global network architecture and form functionally specialized systems that support specific subsets of cognitive tasks or information processing^67–70^. It is important to understand that the methodological approaches and conceptual assumptions employed when running community detection on brain network data affect the community structure outcome. A community detection approach ideally suited for all applications does not exist^14,71^ and the modular, internal density approach represents only one plausible lens through which to view network communities^12,15^. The main contributions of the current study are to demonstrate the usage and utility of a generative modeling approach to community detection in brain network data, with a specific application to measuring changes in structural networks across the human life span.

Here we demonstrate an application of a block modeling approach to community detection in brain networks. The key advantage of this approach is the capacity to parse a brain network into a diverse set of communities^16^; with communities possibly exhibiting modular, core-periphery, or disassortative topologies. In the WSBM consensus partition, we see evidence of this mixed topology. Community *seven* in the model is an example of a disassortative community that modularity maximization would not be able to find. The community, consisting of nodes along the cingulate cortex, is weakly connected within-community and more strongly connected to communities *six* and *ten*. Three WSBM communities, *one*, *four*, and *ten*, have at least one off-diagonal average strength that exceeds the on-diagonal average strength. Importantly, we should note that using the WSBM does not preclude the identification of traditionally modular communities, such as the highly inter-connected nodes of community *seven*, containing nodes of the visual area. Additionally, interesting differences exist between the WSBM and modular partitions. WSBM community *one* contains bilateral prefrontal cortex nodes, whereas the prefrontal nodes of the modular partition are divided between communities *one* and *six*. Community *nine* of the WSBM partition indicated that the bilateral nodes of the PCC and precuneus connected in a stochastically equivalent manner, whereas in the modular partition the precuneus nodes form a small and segregated two-node community. The WSBM community *eight* contained temporal nodes of both hemispheres, whereas the modular community *eight*, contains a large community spanning the right hemisphere.

We measured the extent to which each community structure captured patterns in our brain network data and demonstrated that the WSBM partition represented group averaged and group level data better than the modular partition. Using a generative modeling evaluation framework^4^ we demonstrate that parameters of the WSBM generate synthetic brain networks that deviate less from empirical data than do synthetic brain networks created with parameters estimated from the modular community structure. The WSBM model performed most poorly modeling the clustering coefficient distribution, which is expected given the design of both modular and SBM models and has been confirmed by previous work^38^. In an additional evaluation of how these community structures align with the brain network data, we show that the WSBM partition modeled the symmetry of the brain better than the modular model. The statistical analysis confirms a visually obvious difference between the partitions: the WSBM partition is more symmetrically dispersed across the brain hemispheres than the modular partition.

When evaluating how these community structures change over the life span, we observe that the strength between communities follows inverted-U trends—patterns which align with previous life span studies^7,29,30,33,72^. Here we show that these patterns extend to communities identified with a WSBM approach that covers a much wider space of possible network partitions. We found the strongest age-related change in strength between the nodes of community *three*, which cover the frontal cortex in modularity- as well as WSBM-derived partitions. This finding is in line with previous studies that have shown that ventromedial prefrontal white matter connecting ventromedial prefrontal nodes is particularly vulnerable to aging processes^73,74^. Within-community connectivity of WSBM community *six*, containing bilateral nodes of somatomotor cortex and postcentral cortex, also displays a strong inverted-U quadratic trend (Fig. 6, panel a) that is likely due to age-related changes involving the integrity of corpus callosum connections^31,33,75^. In the modular partition, this trend, which appears at the connection between communities *six* and *ten*, is attenuated (Fig. 6, panel b).

To assess overall community structure changes, we employed vector similarity/distance comparisons. Employing the cosine similarity measure, we observe a pattern where individual subjects maintain similarity to the consensus partition until around the 6^th^ decade of life, where a steep drop-off occurs. This trend could indicate a range of the life span with a stable community structure regardless of connectivity strength, since cosine similarity is a measure of vector orientation but not magnitude. When employing the city block distance, we can then observe a U-shaped trend in distances to the consensus partition, which is likely due in part to connectivity strengths modulated across age. Given that the WSBM partition results in MLR models in which age explains more of the variance in our outcome measures, the WSBM partition appears to be a representative group model for the brain network data across a large age range.

We also fit the WSBM and modular community structures to individual brain networks. This analysis rendered much weaker trends with age; indicating that individually fit community partitions vary substantially from our consensus model and data. We also used these individually fit community structures to analyze the variability of the fit partitions. In panel d of Fig. 7, we report differences in nodal versatility measured across subjects. The difference in nodal versatility maps between the WSBM and modularity maximization methods demonstrates a difference in flexibility between the methods. Recall that the WSBM aggregates nodes with similar connectivity patterns into a community, whereas modularity maximization parses the network into densely connected subnetworks. Unfortunately, the process of detecting modular communities may be influenced by a distance-based bias in the structural brain network data^76^, which results in a spatially compact lateralized community structure. This bias most-likely affects the overall spatial layout of each community structure; we found smaller within-community distance between nodes of the modular than for the WSBM partition. To get closer to the biological reality would likely require several interconnected steps, including a systematic investigation of spatial and/or geometric bias in tractography, how these biases are expressed across age^33^, and how they affect the detection of streamlines and tracts that vary in length, curvature and trajectory^77^. Future work is needed to fully address these challenges.

In the present study, we show how a generative modeling approach to community detection in brain networks might differ (and confer some modeling advantages) compared to a modular approach. However, we would like to reinforce the notion that the choice of community detection algorithm should depend on factors related to the observed data and analytical goals^11^. These two community detection perspectives satisfy differing algorithmic criteria to define communities with different properties^12,15^. A nuanced, but crucial, point to consider is that community structures reflect a plausible grouping of nodes^14^. This organization, sometimes referred to as the *mesoscale* of a brain network^16^, in conjunction with community-based network statistics (such as participation coefficient) can elucidate patterns or trends in a brain network^9,10^. To infer a community structure from brain network data is to parse the data—which can always be trivially organized into some grouping. Whether that organization is biologically and functionally meaningful requires further experimental evidence or metadata^78^. Thus, we would not assert that the WSBM perspective is ‘better’ at capturing the underlying anatomic organization than the modular perspective of communities in brain networks^14^. In fact, we present evidence in the supplemental materials that both algorithmic approaches capture non-random spatial configurations, across parcellation scheme (Fig. S7). The modular approach is certainly valid for network neuroscience applications^5^, and has been employed, for example, to help explain how brain networks might be efficiently embedded in space^69^, to characterize functional MRI during learning^8^ and to differentiate between clinical groups^79^. In the current study, the modular partition does as well as the WSBM partition to capture the block interaction (3–3, Fig. 6) with the highest *R*^2^ value. Additionally, while modularity maximization is designed to consider on-diagonal block interactions, some unmodeled off-diagonal interactions in our evaluation still display statistically significant trends. This considered, recent work has demonstrated a theoretic convergence of the statistical modeling and modularity maximization approaches in special cases of the SBM^80,81^. Future advances along this line of research could better clarify the tradeoffs between inference of SBM and modular partitions.

In the current study, we applied new methods to resolve a consensus model from many community structure solutions of the WSBM inference. Although we measured the consistency of our method, we do note that stochasticity in the current framework still exists. We recognize that there are further parameters of the model that could be optimized, such as prior distribution parameters and parameters governing the convergence criterion for the multiple loops of the variational-Bayes approximation approach. Additionally, we recognize that the WSBM inferred on our data has shortcomings. When using a normal distribution to model edge weights between communities, using the WSBM tools at our disposal we cannot assure that the model will completely avoid modeling negative weights. However, because there are no actual negative edges in our brain network data, we can assume that modeling too many negative edge weights would create lower likelihood, meaning such a model would not be retained by the WSBM inference. Because of this concern, we conducted our generative model analysis with binary network statistics based on edge-existence. Thus, the generative modeling validation could be improved upon by using non-negative weight distributions in future work.

Finally, we note that diffusion imaging and tractography perform computational inference rather than direct measurement of brain connectivity and thus must be interpreted with care^77^. We made efforts in the current study to mitigate against certain biases. We used streamline density as an edge-weight to mitigate against the bias of large regions of interest and we seeded multiple streamlines randomly in each white matter voxel to obtain thorough streamline coverage across the brain. Additionally, we used anatomically-constrained streamline filtering process to recover only streamlines terminating in grey matter^52^. Despite these efforts, future work is needed to further improve the accuracy and sensitivity of structural connectivity measurements derived from noninvasive neuroimaging. In particular, objective quality control metrics can be used increase dMRI data fidelity, which could lead to more accurate associations between dMRI-derived data and age^82^.

In conclusion, we describe a method for applying the WSBM to brain networks, with an application across the life span. We hope to demonstrate the utility of a generative modeling approch to more fully characterize the community structure of brain networks, beyond simple modularity. Our study opens new avenues for using the WSBM for brain network analysis as well as introduces frameworks through which WSBM partitions could be associated with phenotypic characteristics or variations in cognition/behavior^16,78,83^. Future work should use this model to identify how community structure regimes, such as modular, core-periphery, or disassortative models (or a mix of these regimes), relate to aspects of behavior and cognition. Our study shows that the WSBM can provide a flexible and versatile model of brain network community structure and may offer new insights beyond those delivered by modularity analysis.

## Electronic supplementary material

Supplementary Information

## Data Availability

Subject-level adjacency matrices are made availabe at 10.6084/m9.figshare.6983018. Code is available at https://github.com/faskowit/Faskowitz2018wsbmLifeSpan.
