# Supplementary material for: Weighted Stochastic Block Models of the Human Connectome across the Life Span
Source: Sci Rep. 2018 Aug 29;8:12997. doi: 10.1038/s41598-018-31202-1 (PMC6115421; doi:10.1038/s41598-018-31202-1)
Supplement: Supplementary file 1 — Supplementary Information [file 41598_2018_31202_MOESM1_ESM.pdf]

Weighted Stochastic Block Models of the Human Connectome across the Life Span

Joshua Faskowitz[1,2], Xiaoran Yan[3], Xi-Nian Zuo[4,5,6], Olaf Sporns[1,2,3,*]

1. *Program in Neuroscience, Indiana University, Bloomington, IN, USA*
2. *Department of Psychological and Brain Sciences, Indiana University, Bloomington, IN, USA*
3. *Indiana University Network Science Institute, Indiana University, Bloomington, IN, USA*
4. *CAS Key Laboratory of Behavioral Science, Institute of Psychology, Beijing, China*
5. *Research Center for Lifespan Development of Mind and Brain (CLIMB), Institute of Psychology, Beijing, China*
6. *Key Laboratory for Brain and Education Sciences, Nanning Normal University, Nanning, Guangxi 530001, China.*

Supplementary Information

Dataset information

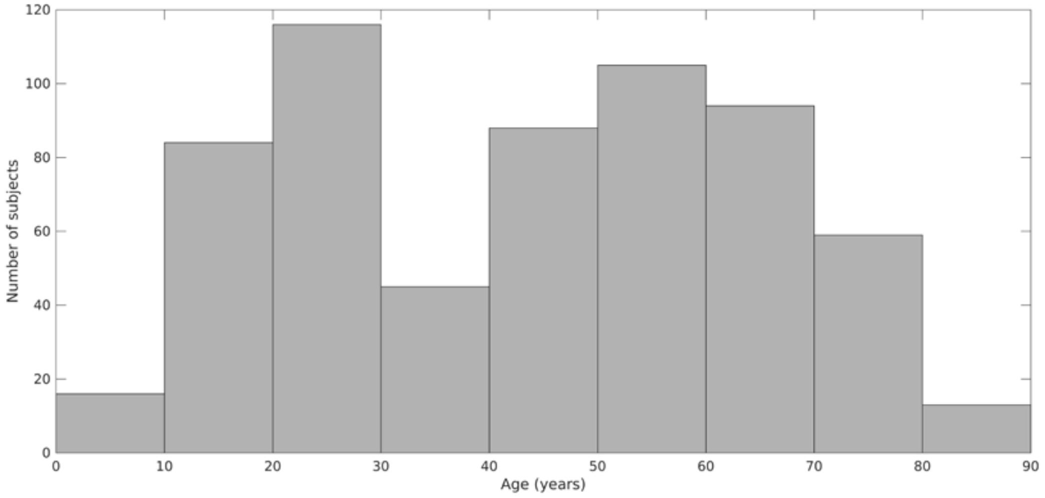

Supplementary Figure S1 A histogram of NKI Rockland sample subject ages; for the 620 subjects used in this study.

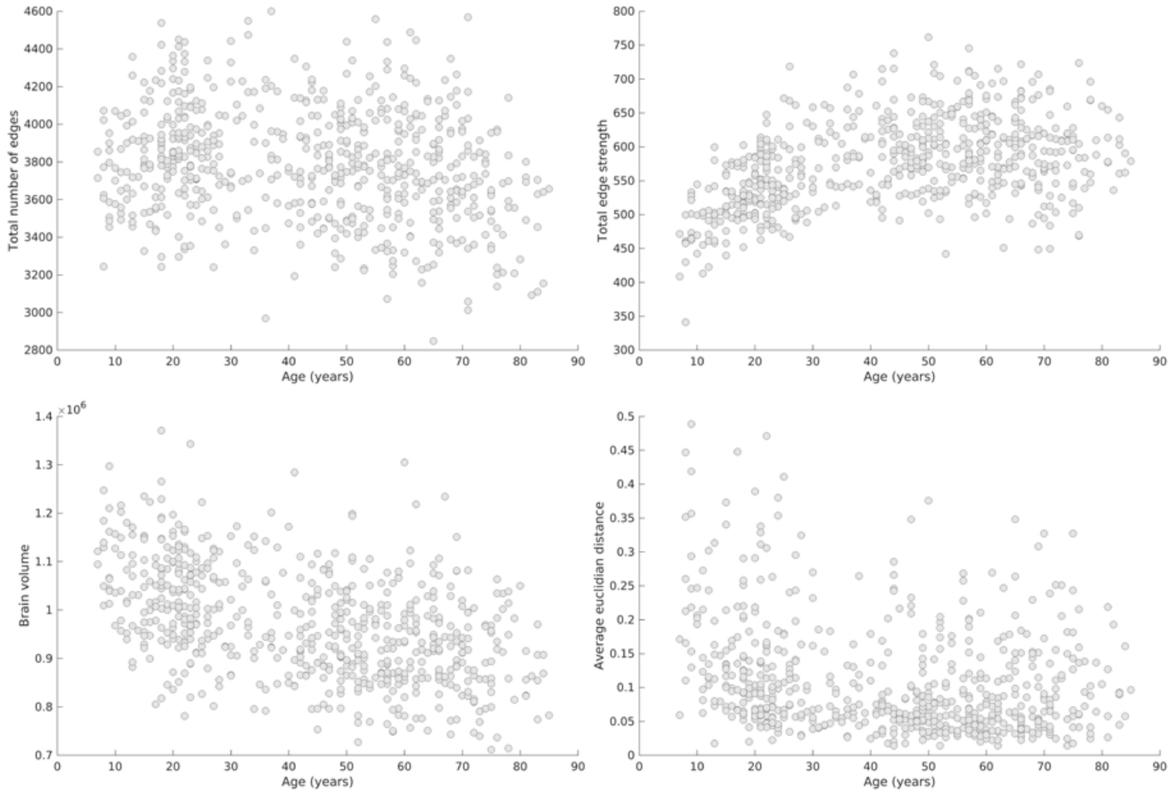

Supplementary Figure S2 Scatterplots of age versus various subject measurements. **Top-Left**: Age versus total number of edges; here we see a slight downward slope which aligns with the reported decrease in number of recovered streamline connections with age [1,2] **Top-Right**: Age versus total edge strength; the sum of all edges weights appears to increase with age. **Bottom-Left**: Age versus brain volume; Supratentorial brain volume measured by FreeSurfer. **Bottom-Right**: Age versus Euclidean distance; the average amount of movement measured after aligning volumes of the dMRI scan. Details to calculate the measure can be found at the following DOI: *10.15200/winn.146228.88496*.

Network measure definitions

Let $A_{i,j}$ be an undirected and weighted adjacency matrix, let the community label of node i be $z_i \in Z$, where $Z = \{1, \dots, k\}$ is a set of k number of community labels. Let r and s be communities $\{r, s\} \in \{1, \dots, k\}$.

Mean within-community & mean between-community strength

The mean within-community (W_{within}) and mean between ($W_{between}$) community strengths are measures of connectivity at the level of community structure.

Let $\mathcal{w}$ be the average block matrix defined as:

$$\mathcal{w}_{rs} = \frac{1}{n_r \cdot n_s} \sum_{i \in r, j \in s} A_{i,j} \ , \qquad \text{if } r \neq s$$

$$w_{rr} = \frac{1}{n_r \cdot (n_r - 1)} \sum\nolimits_{i \in r, j \in r} A_{i,j}$$

Where n_r and n_s are the number of nodes in communities r and s. The mean within-community strength for community r would be:

$$W_{within} = w_{rr}$$

and the between-community for community r would be

$$W_{between} = \frac{1}{k-1} \sum\nolimits_{r \neq s} w_{rs}$$

Participation coefficient

The participation coefficient of node i (P_i) is a measure of how well-connected node i is to is own community versus other communities. If a node is uniformly connected to all communities, the participation coefficient will be near 1. If a node is only connected to nodes of the same community, its participation coefficient will be 0. See [3] equation 4. It is defined as:

$$P_i = 1 - \sum_{r \in Z} \left(\frac{d_i(r)}{d_i} \right)^2$$

where Z is the set of communities, d_i is the weighted degree for node i, and $d_i(r)$ is the weighted degree between i and all nodes in community r.

Community assortativity

Community assortativity [4] for community r ($\mathcal{A}_r$) compares the within community strength, which we can think of as on-diagonal community strength, with the max between community strength, which we can think of as the max off-diagonal community strength. It is defined as:

$$\mathcal{A}_r = \left[w_{rr} - \max_{s \neq r}(w_{rs}) \right]$$

Nodal assortativity

The nodal assortativity [4] for node i (α_i) compares a node's weighted connectivity to its assigned community (z_i) to the maximum weighted connectivity to other communities. Given node i's community assignment z_i, its weighted connection density to community r is defined as

$$a_i(r) = \frac{1}{n_r} \sum\nolimits_{j \in r} A_{ij}$$

then the nodal assortativity can be defined as:

$$\alpha_i = a_i(z_i) - \max_{r \neq z_i} a_i(r)$$

Within-module (community) z-score

The within-module z-score [5] for node i (I_i) is a measure of a node's relative within-community connectivity, given within-community connectivity for all the nodes of the same community. It is defined as:

$$I_i = \frac{d_i(r_i) - \bar{d}_\iota(r_i)}{\sigma^{d(r_i)}}$$

where r_i is the community containing node i, $d_i(r_i)$ is the within-community weighted degree of node i, and $\bar{d}_\iota(r_i)$ and $\sigma^{d(r_i)}$ are the mean and standard deviation of the within-community weighted degree distribution.

Nodal versatility

The nodal versatility [6] for node i (V_i) is an index of how readily a node is assigned a community with the same neighboring nodes. It is defined as:

Where p is the pairwise membership probability, or in other words, the probability of nodes i and j resulting in the same community:

$$p_{i,j} = \frac{\sum_n^{\mathcal{N}} g^n(z_i, z_j)}{\mathcal{N}}$$

where $\mathcal{N}$ is the number of repetitions of a community detection algorithm and the g is the agreement matrix, where:

$$g^n(i,j) = \begin{cases} 1, & \text{if } z_i = z_j \text{ for the } n\text{th repetition} \\ 0, & \text{else} \end{cases}$$

then the nodal versatility for node i is:

$$V_i = \frac{\sum_j \sin(\pi \cdot p_{i,j})}{\sum_j p_{i,j}}$$

Consensus convergence

Consensus convergence [7] ($\mathcal{C}$) is an index of how consistently nodes are assigned the same community membership, given a pairwise membership probability matrix, p. It is defined as:

$$\mathcal{C} = \frac{\sum_{(i,j)\in\mathcal{G}} (p_{i,j} - 0.5)^2}{|\mathcal{G}|} \times \frac{1}{(0.5)^2}$$

where $\mathcal{G}$ is the set of nonzero entries in the agreement matrix, g, and $|\mathcal{G}|$ is the size of set $\mathcal{G}$.

Further workflow validation methods and results

For the generative module evaluation framework, we sought to show how the WSBM consensus model could effectively generate synthetic adjacency matrices. Given that the consensus community structure models were fit to the young adult representative matrix, there is a possibility that the WSBM model could be overfit to the input data. To measure the

generalizability of the model, we measured the energy between generated synthetic networks of our consensus models and each adjacency matrix in our sample. Specifically, for each subject we measured the mean KS energy over 5000 iterations for each consensus model (WSBM and modular). We found that the WSBM consensus model produced lower energies (M=0.4, SD=0.013) on average than the modular consensus model (M=0.42, SD=0.014; $t(1.24x10^3)$=-27.28, $p < 10^{-9}$).

When evaluating the symmetry of our consensus partitions, we wanted to ensure that the symmetry demonstrated by the WSBM is not merely a result of a consensus fit procedure. Therefore, we repeated this hemispheric symmetry analysis 100 times, switching out the consensus WSBM partition for one of the 100 WSBM models of our fitting workflow (the models represented by Figure 2, panel b). These are models fit to the representative adjacency matrix at a given k, but not modified by consensus information. In 97 of the 100 WSBM tested, the partition produced a distribution of hemispheric KS scores (across 620 subjects) significantly lower (Wilcoxon rank sum test; p < 10-9; z-value range for significant comparisons: -25.87 – -8.95) than the distribution of hemispheric KS scores (across 620 subjects) derived from the modular partition.

Supplemental results using a different brain parcellation

In our original analysis, we used a brain parcellation consisting of 114 nodes in the cortical grey matter, derived from a clustering method applied to 1000 resting-state fMRI scans [8]. Many plausible parcellations of the cortical grey matter exists, constructed to optimize a variety of objective functions [9]. It is therefore good practice to perform brain network analysis on an additional parcellation; which is what we do here. For this reevaluation, we chose to use the Lausanne scale125 parcellation (scale125), containing 234 cortical nodes [10]. This parcellation was created by randomly subdividing the nodes of a widely-used structural parcellation [11]. In the same manner as previously, we recorded the streamline density between each region of the scale125 parcellation to create our subject-level data. By changing the number of nodes being used, we also changed the sparsity of the individual-level data, necessitating a new sparsity cutoff which was set at 0.15; yielding 609 subjects to analyze. The young adult representative matrix was created from 50 subjects between 25-35 years old, with an edge-existence density of 17.7%. Following the creation of the young adult representative matrix, we performed the identical analysis as described before (with the Yeo parcellation); however, we did not repeat the individual fit analysis due to computational feasibility. Here we describe these results and find that they align generally with our previous findings.

Using the scale125 node definitions, we identified 11 communities using the WSBM consensus workflow (supplementary Figure S4). Like previously, the WSBM has modeled some off-diagonal block interactions with high edge weights; such as interactions *6-8* and *4-10*. The WSBM consensus model contains 3 communities that we could consider disassortative (communities *4*, *5*, and *10*) while the modular consensus model does not contain any such communities (supplementary table S1). Using MLR to measure strengths between block interactions, we see that in the WSBM partition, the top three MLR trends as measured by R^2 are off-diagonal whereas the top three MRL trends for the modular partition are on the diagonal (supplemental Figure S5).

We again find that the WSBM model generates synthetic brain networks (supplemental Figure S6, panel a) with a lower energy than the modular model ($t(1999)=-21.11$, $p < 10^{-9}$); supplemental Figure S6). However, here we find that both the WSBM and modular models perform considerably better than the randomized counterpart model. When measuring how well each consensus partition respects brain symmetry (supplemental Figure S6, panel b), we find that in contrast to the results reported with the Yeo parcellation, the modular partition has a slightly smaller between hemisphere KS when measuring participation coefficient ($t(1.18x10^3)=4.84$, $p = 1.5 \times 10^{-6}$). The differences observed for within-module z-score and assortativity recapitulate what we previously observed ($p < 10^{-9}$ and $p = 0.0077$, respectively). Finally, measuring vector similarity/distance between individual subjects and the consensus vectors (supplemental Figure S6, panel c) yielded results analogous to the previous findings, the vector measurements given the WSBM partition are more similar and less distant than the vector measurements made using the modular partition ($p < 10^{-9}$ for both). In all tests, without and with covariates, the R^2 values of the WSBM trend is high than the R^2 values of the modular trend.

Comparing community structure partitions across parcellation

We also assessed the performance of community detection algorithm across parcellation choice (Yeo and scale125), to see if the community structure identified by a specific model for one parcellation would be statistically similar to the community structure identified by the same model, but with a difference parcellation. Straightforward computation of a similarity is not feasible as the number of nodes between the parcellations differs. To make this comparison, we obtained the spatial arrangement of the parcellations on the FreeSurfer average surface, which contains 163842 vertices per hemisphere. For each vertex not part of the “medial gap” (the area where the two hemispheres are attached through the callosum and subcortical volumes), we identified the community assignment for that vertex. This resulted in 135679 and 135625 valid vertices for the left and right hemispheres, which were concatenated to create a vector of length 271,304. Projecting the communities onto the same geometric space allowed us to measure the variation of information (VI; distance) and adjusted rand index (ARI; similarity) between the vector of community assignments across parcellation. We computed both of these measurements, to make sure that we could observe converging distance/similarity results [12]. Were measured the empirical distance/similarity between the WSBM partitions in both the Yeo and scale125 parcellations. We repeated this procedure for the modular partitions as well.

To assess statistical significance of these spatial community structure distances/similarities, we employed a spin-based permutation test [13,14]. For each hemisphere of the fsaverage surface, we have a mapping of the points in a spherical space (Figure S7, panel a). For each permutation, and repeated within an iteration for each hemisphere, a sphere is randomly rotated along the x, y, and z axes. Based on this rotation, spatial map originally in the corrected fsaverage space can be transformed to new points along the surface. The advantage of such a permutation method is the ability to maintain the spatial adjacencies of the parcellation. We performed 5000 spin permutations and recorded the VI and ARI between the unrotated Yeo, and the rotated scale125 at each permutation, disregarding the medial gap areas. At each iteration, the WSBM scale125 and the modular scale125 partitions were rotated according to the same angles.

We found that for both the WSBM and modular community structures, the identified partitions are less distant than by chance. We also see that the modular community structures across parcellation are less distant than the WSBM community structures. Overall, this analysis shows that the spatial similarity within algorithm (and across parcellation) is significant, for both methods. The empirical VI and ARI are outside the range of null values (empirical VI is to the left of the null distribution, empirical ARI is to the right of the null distribution) for each test (Figure S7). How should we interpret the finding that the modular partitions appear “closer” across the two parcellations than the WSBM partitions? We do not think that this necessarily indicates greater robustness on the part of modular partitions. An alternative interpretation is that modular partitions more strongly reflect spatial (proximity) effects (that are independent of partitions).

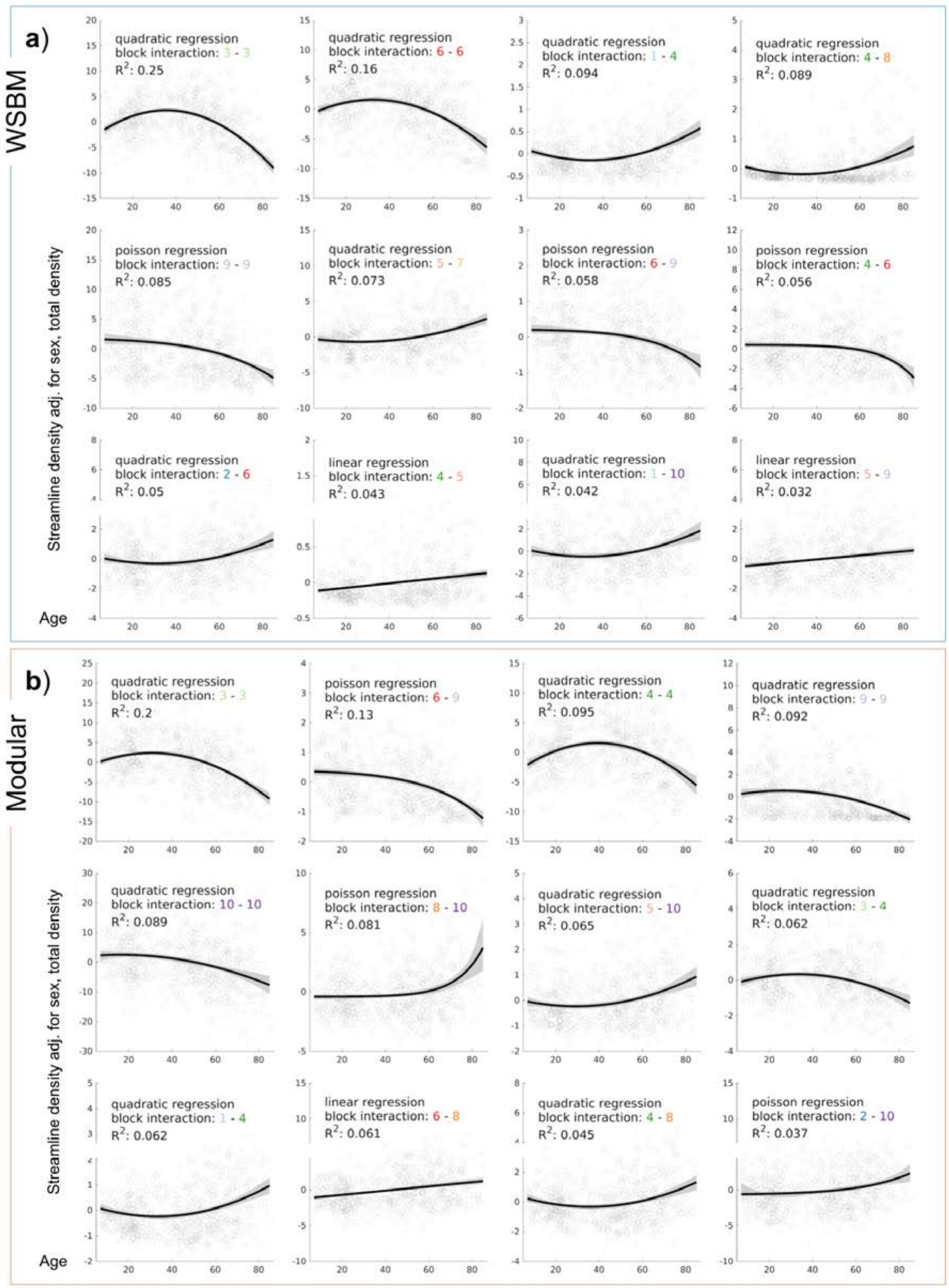

Supplemental Figure S3 Extended results of multiple linear regression analysis on edge strengths of community interactions in the WSBM (**a**) and modular community (**b**) structures; here we visualize the top 12 MLR trends for both community structures. The top three trends in both (**a**) and (**b**) are repeated from Figure 6 in the main text.

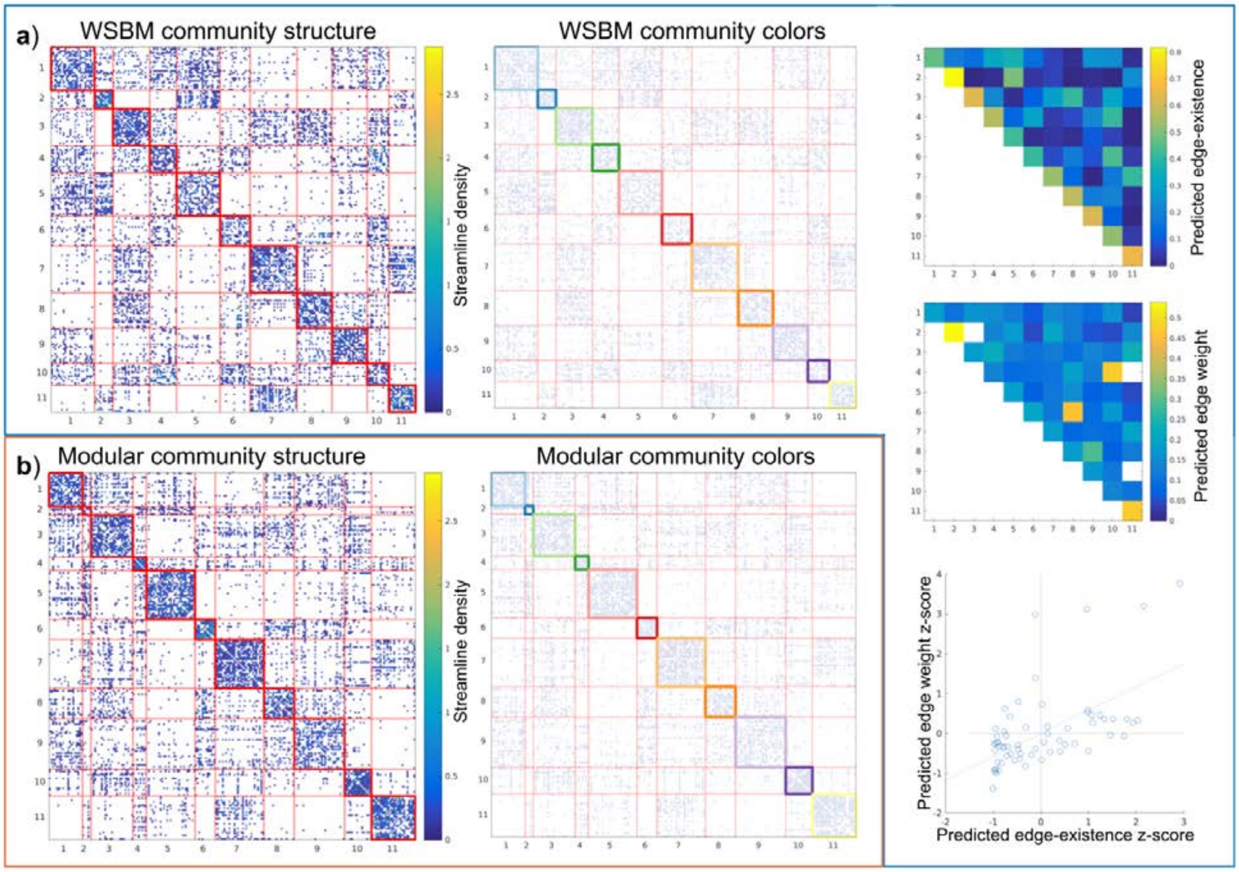

Supplementary Figure S4 WSBM and modular consensus models fit to a representative matrix averaged across 53 young adult subjects, using nodes from the Lausanne scale125 parcellation (214 cortical nodes). Note that the colors of communities in this figure do not correspond to the colors used in the analysis using the Yeo parcellation. **a)** The analogous figures to Figure 3 of the main text are shown. From top left to bottom right: the adjacency matrix ordered by the blocks of the WSBM consensus model; the community structure of the WSBM visualized with colors; the predicted edge-existence and edge weight matrices (white entries in the predicted edge weight matrix indicate where a negligible probability of an edge existing is modeled); the paired parameters of the block interactions (z-score transformed). **b)** The adjacency matrix ordered by the modular consensus model and community structure of the modular model visualized with colors.

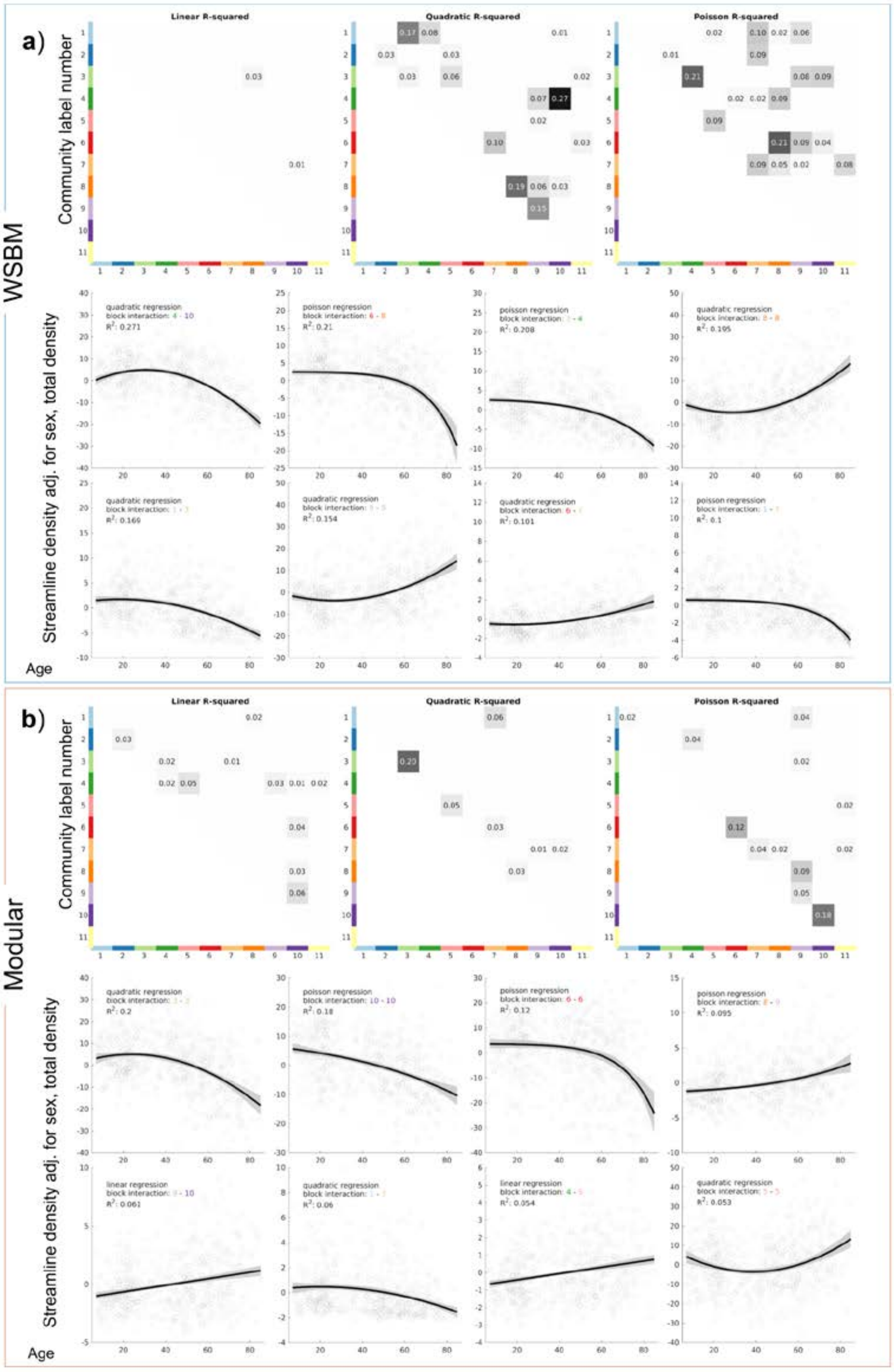

Supplementary Figure S5 Results of multiple linear regression analysis on edge strengths of community interactions in the WSBM (**a**) and modular community (**b**) structures for the Lausanne scale125 parcellation. Note for this figure, we display all trends with R^2 values above 0, instead of thresholding based on p-value.

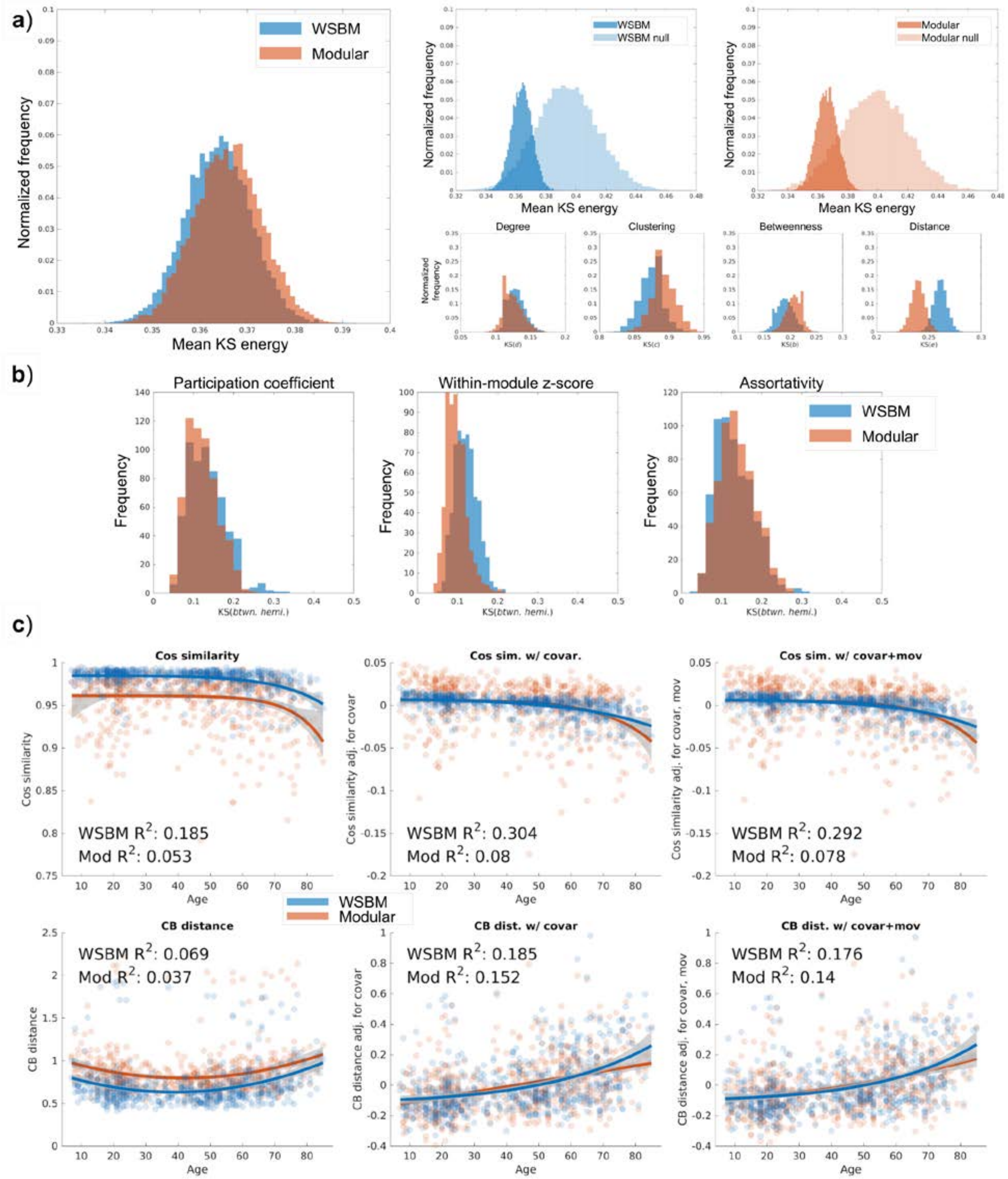

Supplementary Figure S6 Figures for analogous analysis conducted when using the nodes of the Lausanne scale125 parcellation. **a)** Evaluation of generative energy between WSBM and modular models **b)** Evaluation of between hemisphere KS statistics given WSBM or modular structure **c)** Evaluation of static community structure vector similarity/distance.

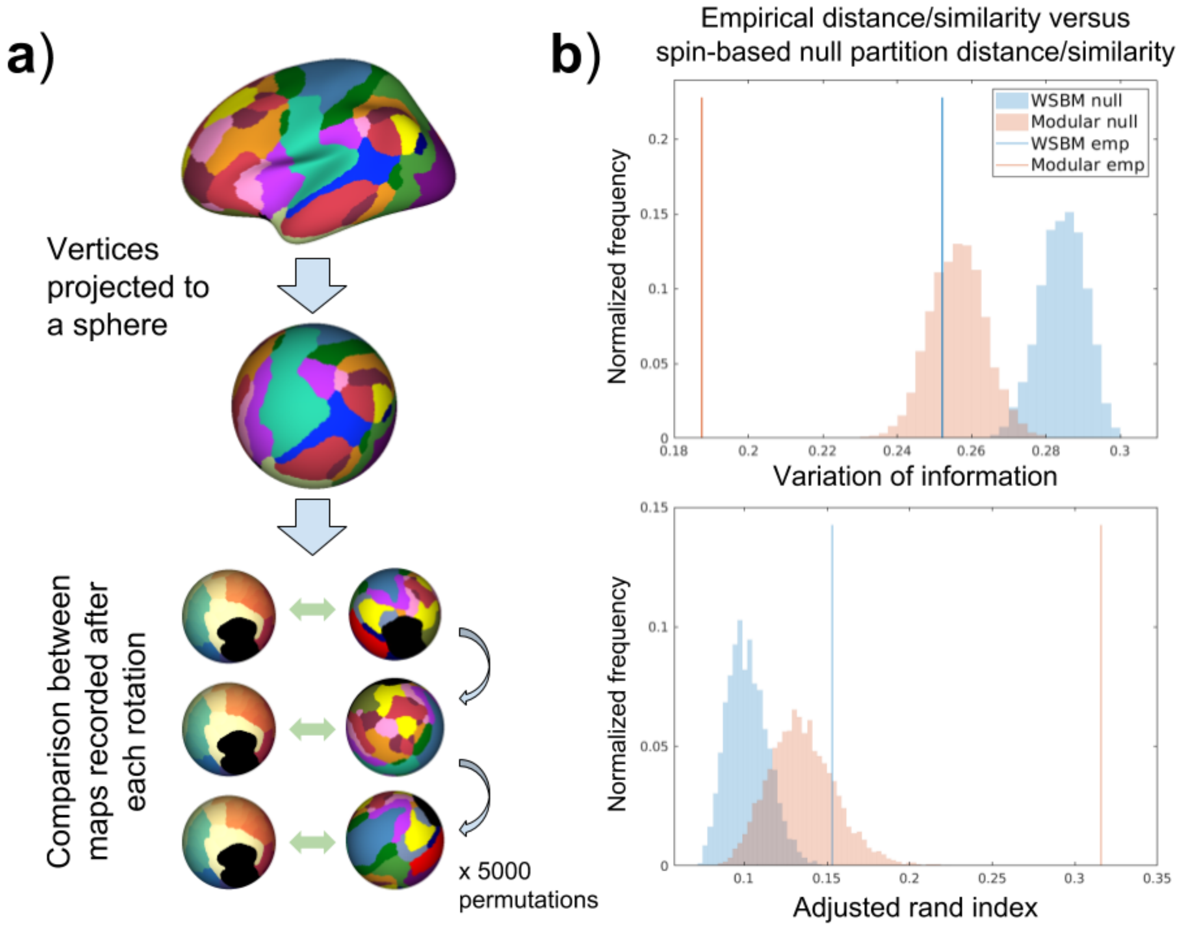

Supplemental Figure S7 a) An illustration of the surfaces used in spin-based permutation test; the FreeSurfer fsaverage surface vertices can be transformed to a sphere and in the space of the sphere, random rotations in the x, y, and z planes can be applied to create null parcellations; colored with Yeo and scale125 parcellation for illustrative purposes **b**) Results of spin-based permutation test when comparing the WSBM partitions of the Yeo and scale125 parcellations (blue), and when comparing the modular partitions of the Yeo and scale125 parcellations (orange); lines represent the empirical measurements and lighter shade distributions reflect null measurements; for both partitions, and for both variation of information and adjusted rand index, the empirical measurement is out of the range of the null distribution

Supplementary Table S1 Table of community statistics for the WSBM and modular consensus partitions using the Lausanne scale125 parcellation. Statistics from the representative young adult matrix; across-subject mean ± standard deviation in parentheses

Community Label	Mean within-community strength	Mean between-community strength	Mean community participation coef.	Community assortativity
WSBM				
1	0.09 (0.083 ± 0.02)	0.022 (0.019 ± 0.0039)	0.75 (0.67 ± 0.027)	0.039 (0.03 ± 0.017)
2	0.44 (0.43 ± 0.083)	0.016 (0.016 ± 0.0035)	0.56 (0.5 ± 0.05)	0.35 (0.36 ± 0.081)
3	0.11 (0.1 ± 0.028)	0.023 (0.019 ± 0.0045)	0.77 (0.67 ± 0.037)	0.066 (0.051 ± 0.024)
4	0.079 (0.07 ± 0.023)	0.038 (0.033 ± 0.008)	0.78 (0.72 ± 0.026)	-0.11 (-0.089 ± 0.054)
5	0.083 (0.08 ± 0.015)	0.017 (0.016 ± 0.0031)	0.65 (0.59 ± 0.032)	-0.0057 (0.0037 ± 0.02)
6	0.086 (0.082 ± 0.019)	0.023 (0.019 ± 0.0041)	0.69 (0.62 ± 0.031)	0.00096 (0.0075 ± 0.018)
7	0.096 (0.088 ± 0.017)	0.018 (0.018 ± 0.0032)	0.65 (0.6 ± 0.029)	0.013 (0.011 ± 0.017)
8	0.11 (0.1 ± 0.033)	0.023 (0.016 ± 0.004)	0.68 (0.55 ± 0.046)	0.022 (0.037 ± 0.036)
9	0.11 (0.097 ± 0.031)	0.014 (0.0096 ± 0.0028)	0.65 (0.54 ± 0.049)	0.052 (0.065 ± 0.03)
10	0.072 (0.07 ± 0.021)	0.04 (0.035 ± 0.0079)	0.76 (0.71 ± 0.028)	-0.12 (-0.089 ± 0.052)
11	0.31 (0.3 ± 0.05)	0.015 (0.015 ± 0.0034)	0.48 (0.46 ± 0.044)	0.23 (0.22 ± 0.047)
Modular				
1	0.11 (0.1 ± 0.025)	0.021 (0.018 ± 0.0042)	0.72 (0.66 ± 0.027)	0.062 (0.05 ± 0.028)
2	0.2 (0.19 ± 0.1)	0.026 (0.025 ± 0.0074)	0.8 (0.73 ± 0.044)	0.14 (0.12 ± 0.095)
3	0.15 (0.12 ± 0.029)	0.021 (0.018 ± 0.0043)	0.66 (0.62 ± 0.032)	0.11 (0.081 ± 0.025)
4	0.22 (0.22 ± 0.086)	0.021 (0.02 ± 0.0056)	0.79 (0.73 ± 0.046)	0.17 (0.15 ± 0.077)
5	0.16 (0.15 ± 0.024)	0.014 (0.014 ± 0.003)	0.42 (0.41 ± 0.041)	0.11 (0.098 ± 0.025)
6	0.39 (0.32 ± 0.11)	0.015 (0.012 ± 0.003)	0.56 (0.53 ± 0.061)	0.33 (0.27 ± 0.11)
7	0.11 (0.1 ± 0.021)	0.017 (0.015 ± 0.0039)	0.59 (0.54 ± 0.045)	0.078 (0.063 ± 0.021)
8	0.17 (0.12 ± 0.035)	0.017 (0.014 ± 0.0036)	0.69 (0.65 ± 0.033)	0.11 (0.068 ± 0.03)
9	0.09 (0.077 ± 0.018)	0.016 (0.014 ± 0.0031)	0.64 (0.58 ± 0.036)	0.052 (0.046 ± 0.015)
10	0.21 (0.17 ± 0.042)	0.017 (0.017 ± 0.0051)	0.64 (0.59 ± 0.059)	0.17 (0.13 ± 0.042)
11	0.21 (0.19 ± 0.029)	0.01 (0.011 ± 0.0025)	0.4 (0.39 ± 0.041)	0.17 (0.15 ± 0.027)

Supplementary Table S2 Node names and community affiliations for WSBM and modular consensus partitions; using the Yeo 17 subdivided nodes. Colors correspond to the label colors for communities used in Figure 3.

WSBM community	WSBM node name	Modular community	Modular node name
1	LH SalVentAttnA Ins	1	LH SalVentAttnA Ins
1	LH SalVentAttnB PFCl	1	LH SalVentAttnB PFCv
1	LH SalVentAttnB PFCv	1	LH ContB PFClv
1	LH Limbic OFC	1	LH DefaultB Temp
1	LH ContA PFClv	1	LH DefaultB PFCv
1	LH ContB PFCl	2	LH SomMotB S2
1	LH ContB PFClv	2	LH SomMotB Ins
1	LH DefaultA PFCd	2	LH SomMotB Aud
1	LH DefaultB Temp	2	LH DorsAttnB TempOcc
1	LH DefaultB PFCv	2	LH DorsAttnB PostC
1	RH SalVentAttnA Ins	2	LH SalVentAttnA ParOper
1	RH SalVentAttnB PFCl	2	LH SalVentAttnA PrCv
1	RH SalVentAttnB PFClv	2	LH SalVentAttnB IPL
1	RH SalVentAttnB PFCv	2	LH ContA IPS
1	RH Limbic OFC	2	LH ContA PFCd
1	RH ContB PFClv	2	LH ContA PFCl
1	RH DefaultA PFCd	2	LH ContB IPL
1	RH DefaultB PFCv	2	LH ContB PFCd
2	LH SomMotB S2	2	LH DefaultA IPL
2	LH SalVentAttnA ParOper	2	LH DefaultB IPL
2	LH SalVentAttnB IPL	2	LH DefaultB PFCl
2	LH ContB IPL	2	LH TempPar
2	RH SomMotB S2	3	LH SalVentAttnB PFCl
2	RH SalVentAttnA ParOper	3	LH ContA PFClv
2	RH SalVentAttnB IPL	3	LH ContB PFCl
2	RH ContA IPS	3	LH DefaultA PFCd
2	RH ContB IPL	3	LH DefaultB PFCd
3	LH ContA PFCl	3	RH SalVentAttnB PFCl
3	LH DefaultA PFCm	3	RH ContA PFCl
3	LH DefaultB PFCd	3	RH ContB PFCld
3	LH DefaultB PFCl	3	RH DefaultA PFCd
3	RH ContA PFCl	3	RH DefaultB PFCd
3	RH ContB PFCld	4	LH SalVentAttnB PFCmp
3	RH DefaultA PFCm	4	LH ContB PFCmp
3	RH DefaultB PFCd	4	LH DefaultA PFCm
4	LH ContA Cinga	4	RH SalVentAttnB PFCmp
4	LH ContC Cingp	4	RH SalVentAttnB Cinga
4	LH DefaultC Rsp	4	RH ContB PFCmp
4	RH SalVentAttnB Cinga	4	RH DefaultA PCC
4	RH ContA Cinga	4	RH DefaultA PFCm
4	RH ContC Cingp	5	LH VisCent Striate
4	RH DefaultC Rsp	5	LH VisCent ExStr
5	LH DorsAttnA TempOcc	5	LH DorsAttnA TempOcc
5	LH DorsAttnA ParOcc	5	LH DorsAttnA ParOcc
5	LH Limbic TempPole	5	LH SalVentAttnB OFC
5	LH DefaultC IPL	5	LH Limbic TempPole
5	RH DorsAttnA TempOcc	5	LH Limbic OFC
5	RH DorsAttnA ParOcc	5	LH ContA Temp
5	RH Limbic TempPole	5	LH ContB Temp
5	RH DefaultA Temp	5	LH DefaultC IPL
5	RH DefaultB AntTemp	5	LH DefaultC PHC
5	RH DefaultC IPL	6	LH DorsAttnA SPL
6	LH SomMotA	6	RH SomMotB S2
6	LH DorsAttnB PostC	6	RH DorsAttnA SPL
6	LH SalVentAttnA FrMed	6	RH DorsAttnB PostC
6	RH SomMotA	6	RH SalVentAttnA ParOper

6	RH DorsAttnB PostC	6	RH ContA IPS
6	RH SalVentAttnA FrMed	7	LH VisPeri Striate
7	LH VisCent Striate	7	LH VisPeri ExStrInf
7	LH VisCent ExStr	7	LH VisPeri ExStrSup
7	LH VisPeri Striate	7	LH DefaultC Rsp
7	LH VisPeri ExStrInf	7	RH VisCent Striate
7	LH VisPeri ExStrSup	7	RH VisCent ExStr
7	LH SalVentAttnB OFC	7	RH VisPeri Striate
7	LH DefaultC PHC	7	RH VisPeri ExStrInf
7	RH VisCent Striate	7	RH VisPeri ExStrSup
7	RH VisCent ExStr	7	RH DefaultC Rsp
7	RH VisPeri Striate	7	RH DefaultC PHC
7	RH VisPeri ExStrInf	8	RH SomMotB Ins
7	RH VisPeri ExStrSup	8	RH SomMotB Aud
7	RH DefaultC PHC	8	RH DorsAttnA TempOcc
8	LH SomMotB Aud	8	RH DorsAttnA ParOcc
8	LH DorsAttnB TempOcc	8	RH DorsAttnB TempOcc
8	LH ContA Temp	8	RH SalVentAttnA Ins
8	LH ContB Temp	8	RH SalVentAttnB IPL
8	LH DefaultA IPL	8	RH SalVentAttnB PFClv
8	LH DefaultB IPL	8	RH SalVentAttnB PFCv
8	LH TempPar	8	RH Limbic TempPole
8	RH SomMotB Ins	8	RH Limbic OFC
8	RH SomMotB Aud	8	RH ContA Temp
8	RH DorsAttnB TempOcc	8	RH ContB Temp
8	RH ContA Temp	8	RH ContB IPL
8	RH ContB Temp	8	RH ContB PFClv
8	RH DefaultA IPL	8	RH DefaultA Temp
8	RH DefaultB Temp	8	RH DefaultA IPL
8	RH TempPar	8	RH DefaultB Temp
9	LH DorsAttnA SPL	8	RH DefaultB AntTemp
9	LH ContA IPS	8	RH DefaultB PFCv
9	LH ContC pCun	8	RH DefaultC IPL
9	LH DefaultA PCC	8	RH TempPar
9	RH DorsAttnA SPL	9	LH ContC pCun
9	RH ContC pCun	9	RH ContC pCun
9	RH DefaultA PCC	10	LH SomMotA
10	LH SomMotB Cent	10	LH SomMotB Cent
10	LH SomMotB Ins	10	LH DorsAttnB FEF
10	LH DorsAttnB FEF	10	LH DorsAttnB PrCv
10	LH DorsAttnB PrCv	10	LH SalVentAttnA ParMed
10	LH SalVentAttnA PrCv	10	LH SalVentAttnA FrMed
10	LH SalVentAttnA ParMed	10	LH SalVentAttnB PFCd
10	LH SalVentAttnB PFCd	10	LH ContA Cinga
10	LH SalVentAttnB PFCmp	10	LH ContC Cingp
10	LH ContA PFCd	10	LH DefaultA PCC
10	LH ContB PFCd	10	RH SomMotA
10	LH ContB PFCmp	10	RH SomMotB Cent
10	RH SomMotB Cent	10	RH DorsAttnB FEF
10	RH DorsAttnB FEF	10	RH DorsAttnB PrCv
10	RH DorsAttnB PrCv	10	RH SalVentAttnA PrC
10	RH SalVentAttnA PrC	10	RH SalVentAttnA PrCv
10	RH SalVentAttnA PrCv	10	RH SalVentAttnA ParMed
10	RH SalVentAttnA ParMed	10	RH SalVentAttnA FrMed
10	RH SalVentAttnB PFCd	10	RH SalVentAttnB PFCd
10	RH SalVentAttnB PFCmp	10	RH ContA PFCd
10	RH ContA PFCd	10	RH ContA Cinga
10	RH ContB PFCmp	10	RH ContC Cingp

Supplementary References

1 Lim, S., Han, C. E., Uhlhaas, P. J. & Kaiser, M. Preferential detachment during human brain development: age- and sex-specific structural connectivity in diffusion tensor imaging (DTI) data. *Cereb Cortex* **25**, 1477-1489, https://doi.org/10.1093/cercor/bht333 (2015).

2 Betzel, R. F. *et al.* Changes in structural and functional connectivity among resting-state networks across the human lifespan. *Neuroimage* **102 Pt 2**, 345-357, https://doi.org/10.1016/j.neuroimage.2014.07.067 (2014).

3 Guimera, R. & Nunes Amaral, L. A. Functional cartography of complex metabolic networks. *Nature* **433**, 895-900, https://doi.org/10.1038/nature03288 (2005).

4 Betzel, R. F., Medaglia, J. D. & Bassett, D. S. Diversity of meso-scale architecture in human and non-human connectomes. *Nat Commun* **9**, 346, https://doi.org/10.1038/s41467-017-02681-z (2018).

5 Rubinov, M. & Sporns, O. Complex network measures of brain connectivity: uses and interpretations. *Neuroimage* **52**, 1059-1069, https://doi.org/10.1016/j.neuroimage.2009.10.003 (2010).

6 Shinn, M. *et al.* Versatility of nodal affiliation to communities. *Sci Rep* **7**, 4273, https://doi.org/10.1038/s41598-017-03394-5 (2017).

7 Kwak, H., Choi, Y., Eom, Y.-H., Jeong, H. & Moon, S. in *Proceedings of the 9th ACM SIGCOMM conference on Internet measurement conference.* 301-314 (ACM).

8 Yeo, B. T. *et al.* The organization of the human cerebral cortex estimated by intrinsic functional connectivity. *J Neurophysiol* **106**, 1125-1165, https://doi.org/10.1152/jn.00338.2011 (2011).

9 Sotiropoulos, S. N. & Zalesky, A. Building connectomes using diffusion MRI: why, how and but. *NMR Biomed*, e3752-n/a, https://doi.org/10.1002/nbm.3752 (2017).

10 Hagmann, P. *et al.* Mapping the structural core of human cerebral cortex. *PLoS Biol* **6**, e159, https://doi.org/10.1371/journal.pbio.0060159 (2008).

11 Desikan, R. S. *et al.* An automated labeling system for subdividing the human cerebral cortex on MRI scans into gyral based regions of interest. *Neuroimage* **31**, 968-980, https://doi.org/10.1016/j.neuroimage.2006.01.021 (2006).

12 Gates, A. J., Wood, I. B., Hetrick, W. P. & Ahn, Y.-Y. On comparing clusterings: an element-centric framework unifies overlaps and hierarchy. Preprint at https://arxiv.org/abs/1706.06136 (2017).

13 Alexander-Bloch, A. F. *et al.* On testing for spatial correspondence between maps of human brain structure and function. *Neuroimage* **178**, 540-551, https://doi.org/10.1016/j.neuroimage.2018.05.070 (2018).

14 Arslan, S. *et al.* Human brain mapping: A systematic comparison of parcellation methods for the human cerebral cortex. *Neuroimage* **170**, 5-30, https://doi.org/10.1016/j.neuroimage.2017.04.014 (2018).
